# Sparse Canonical Correlation Analysis for Multiple Measurements With Latent Trajectories

**DOI:** 10.1002/bimj.70090

**Published:** 2025-10-30

**Authors:** Nuria Senar, Aeilko H. Zwinderman, Michel H. Hof

**Affiliations:** ^1^ Department of Epidemiology & Data Science Amsterdam School of Public Health Amsterdam UMC Amsterdam The Netherlands

**Keywords:** canonical correlation analysis, dimension reduction, high‐dimensional data, repeated measurements

## Abstract

Canonical correlation analysis (CCA) is a widely used multivariate method in omics research for integrating high‐dimensional datasets. CCA identifies hidden links by deriving linear projections of observed features that maximally correlate datasets. An important requirement of standard CCA is that observations are independent of each other. As a result, it cannot properly deal with repeated measurements. Current CCA extensions dealing with these challenges either perform CCA on summarized data or estimate correlations for each measurement. While these techniques factor in the correlation between measurements, they are suboptimal for high‐dimensional analysis and exploiting this data's longitudinal qualities. We propose a novel extension of sparse CCA that incorporates time dynamics at the latent variable level through longitudinal models. This approach addresses the correlation of repeated measurements while drawing latent paths, focusing on dynamics in the correlation structures. To aid interpretability and computational efficiency, we implement an $\ell_{0}$ penalty to enforce fixed sparsity levels. We estimate these trajectories fitting longitudinal models to the low‐dimensional latent variables, leveraging the clustered structure of high‐dimensional datasets, thus exploring shared longitudinal latent mechanisms. Furthermore, modeling time in the latent space significantly reduces computational burden. We validate our model's performance using simulated data and show its real‐world applicability with data from the Human Microbiome Project. This application highlights the model's ability to handle high‐dimensional, sparsely, and irregularly observed data. Our CCA method for repeated measurements enables efficient estimation of canonical correlations across measurements for clustered data. Compared to existing methods, ours substantially reduces computational time in high‐dimensional analyses as well as provides longitudinal trajectories that yield interpretable and insightful results.

## 1 Introduction

Biomedical data, such as genetic or methylation data, are often high‐dimensional, with the number of features far exceeding the number of samples. Analyzing this type of data requires methods capable of addressing challenges posed by high dimensionality, including collinearity and interpretability. Advances in technology have improved the ability to collect high‐throughput biomedical data through enhanced measurement instruments and reduced patient burden, enabling the collection of multiple measurements from the same individual. These multiple (or repeated) measurements can provide valuable insights into the time dynamics of the underlying mechanisms, revealing longitudinal associations that might otherwise remain hidden. These associations offer a deeper understanding of biological or clinical processes. However, repeated measurements from the same patient often introduce dependencies across observations. Furthermore, the data may include missing measurements for some individuals or be recorded at uneven intervals, resulting in sparsely and irregularly observed data.

Integrative methods, such as canonical correlation analysis (CCA), combine multiple datasets through maximally correlated linear projections, typically referred to as latent vectors. By uncovering shared variation between datasets, CCA provides insights into the underlying relations that exist between the variables from these data sources. Sparse extensions of CCA further enhance this exploration by identifying the most relevant variables, helping to mitigate collinearity and focus on key contributors to the observed associations.

To deal with repeated measurements in CCA, Hao et al. (2017) and Du et al. (2019) proposed to estimate a separate sparse CCA for each unique time point, using *fused*‐type penalties to ensure consistency in variable selection across time points. Although these approaches are suitable for high‐dimensional data, they require correspondence and regularity between measurements across datasets. Alternatively, Lee et al. (2023) proposed performing CCA on summarized data for each measurement, effectively addressing irregularly and sparsely observed data, yet remaining unsuitable for high‐dimensional data. The above methods do not focus on the dynamics of latent mechanisms and allow the canonical weights to vary across measurements, which may introduce challenges, such as feedback between the canonical weights and latent variables that may lead to nonidentifiability of their separate contributions. This, in turn, leaves the shared underlying dynamics of correlated feature groups unexplored. Overall, these methods either fail to leverage the longitudinal structure of the data, cannot handle high dimensionality, or are limited by irregularly observed measurements.

We address all three points above modeling the longitudinal dimension in the latent space while keeping the canonical weights constant across measurements thus tracking the evolution of these correlated underlying mechanisms. For that, we keep the canonical weights fixed across measurements, treating the latent variables as dynamic trough time. Since the latent space is low‐dimensional, this approach maintains low computational times, as we estimate the longitudinal dynamics of the latent variables, and not the original observations. To the best of our knowledge, no existing methods focus on describing latent trajectories connecting high‐dimensional datasets.

We validated our model using simulations, where it successfully identified the simulated latent trajectories and the main contributors. Additionally, we applied our method to real data from the Human Microbiome Project (HMP) (Zhou et al. 2019), uncovering latent paths that link gene expression and operational taxonomic units (OTUs) from gut microbiome data for insulin‐sensitive (IS) and insulin‐resistant (IR) patients. Notably, our algorithm described latent paths consistent with findings reported in existing literature.

## 2 Methods

Suppose that we have data matrices $X={\left(X_{1},X_{2},\text{…}X_{n}\right)}^{⊺}$ and $Y={\left(Y_{1},Y_{2},\text{…}Y_{n}\right)}^{⊺}$ containing the repeated measurements from n individuals stacked in long format. Let $X_{i}\in R^{m_{i}\times p}$ be the observations for individual i consisting of $m_{i}$ measurements of p features. Similarly, $Y_{i}\in R^{s_{i}\times q}$ is the observation matrix of $s_{i}$ measurements of q features for i. The times at which the measurements from individual i are obtained are given by $t_{x,i}\in R^{m_{i}}$ for $X_{i}$ and $t_{y,i}\in R^{s_{i}}$ for $Y_{i}$. We denote t to the vector of all measurements if both X and Y such that $t={⋃}_{i}\left(t_{x,i}\cup t_{y,i}\right)$.

**FIGURE 1:**
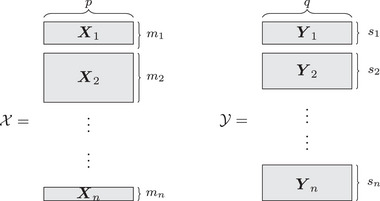
Data structure. The data are composed of n stacked data matrices of p and q features, respectively. The gray rectangles represent the multiple measures, $m_{i}$ and $s_{i}$ for each dataset, collected for each patient i. That is, for X, the each rectangle is $X_{i}\in R^{m_{i}\times p}$ and $Y_{i}\in R^{s_{i}\times q}$ for Y.

Importantly, the number of measurements from individual i and their corresponding collection dates can differ between both data matrices. For instance, individual i may have three measurements in X and five measurements in Y, that is, $m_{i}=3$ and $s_{i}=5$. Additionally, these measurements may have been collected at different moments, for example, $t_{x,i}=\left(1,3,4\right)$ and $t_{y,i}=\left(1,2,3,4,6\right)$, which leaves $t_{i}=\left(1,2,3,4,6\right)$. Moreover, these vectors may be organized to represent different time frames or sequences, depending on the longitudinal path of interest. For instance, these can simply indicate measurement number (as in the above example), collection dates or dates relative to an event of interest, as shown in Section 3.2. This data structure is represented in Figure 1.

The main objective of CCA is to extract K pairs of latent variables $\left\{\left(\eta_{1},\gamma_{1}\right),\left(\eta_{2},\gamma_{2}\right),\text{…},\left(\eta_{K},\gamma_{K}\right)\right\}$ from both data matrices. Each latent variable is a linear combination of the features from a particular data matrix, that is, the kth pair of latent variables for individual i is given by $\eta_{k}=Xw_{x,k}$ and $\gamma_{k}=Yw_{y,k}$, where the weights $w_{x,k}\in R^{p}$ and $w_{y,k}\in R^{q}$ denote the contribution of each variable.

The weights $W_{x}=\left(w_{x,1},\text{…},w_{x,K}\right)$ and $W_{y}=\left(w_{x,1},\text{…},w_{x,K}\right)$ are chosen such that the canonical correlation between all pairs of latent variable is maximized, that is,

(1) $$\begin{array}{cc} ({\hat{W}}_{x},{\hat{W}}_{y})= & \arg\min_{W_{x},W_{y}}\sum_{i=1}^{n}\left||X_{i}W_{x}-Y_{i}W_{y}\right|{|}_{2}^{2} \end{array}$$

under the restriction that the columns in the sets $\left(\gamma_{1},\text{…},\gamma_{K}\right)$ and $\left(\eta_{1},\text{…},\eta_{K}\right)$ are orthogonal.

In this paper, we focus on the thresholded ordered sparse CCA (TOSCCA) Senar et al. (2024) algorithm to obtain sparse weights for both datasets. Note, however, that our extension is general and can be applied to other CCA estimation procedures.

### 2.1 Time Dynamics

Since the number and timing of measurements for each individual can differ, we propose to add the longitudinal nature of the data to the CCA objective by assuming that the time dynamics only directly affect the shared hidden path. That is, the longitudinal trajectories affect the observations only through latent variables. Modeling the time dynamics in the latent space will not only help interpretation of the model, it also keeps the computation burden low, as the latent space is low‐dimensional. We assume weights $W_{x}$ and $W_{y}$ to be fixed over time. Based on both assumptions, for each component k=1,⋯,K, the observation from the ith individual from dataset X at time t∈R is a p‐dimensional vector that follows

(2) $$\begin{array}{cc} x_{i}\left(t\right) & =\eta_{i}\left(t\right)W_{x}^{⊺}+\varepsilon_{x,i}\left(t\right)\\ & =f\left(t;\theta_{\eta }\right)W_{x}^{⊺}+\varepsilon_{x,i}\left(t\right), \end{array}$$

where $\varepsilon_{x,i}\left(t\right)$ is white noise.

Similarly, the observation at time t∈R for individual i from dataset Y is a q‐dimensional vector that can be described by

(3) $$\begin{array}{cc} y_{i}\left(t\right) & =\gamma_{i}\left(t\right)W_{y}^{⊺}+\varepsilon_{y,i}\left(t\right)\\ & =f\left(t;\theta_{\gamma }\right)W_{y}^{⊺}+\varepsilon_{y,i}\left(t\right), \end{array}$$

where $\varepsilon_{y,i}\left(t\right)$ is white noise.

The function f(·) describes the underlying mechanism linking both sets of observations through time. We assume this mechanism to follow the same model for both datasets. This is in line with the probabilistic CCA (Bach and Jordan), where a single latent variable generates both datasets. Even though we separate this latent variable into two, η and γ, the same principle applies.

For instance, f(·) may follow a linear mixed effect (LME) model with random intercepts and fixed effects for time for the latent trajectories, that is, $f\left(t;\theta_{\eta }\right)=\alpha_{x,i}+\beta_{x}t$ and $f\left(t;\theta_{\gamma }\right)=\alpha_{y,i}+\beta_{y}t$, where both $\alpha_{x,i}$ and $\alpha_{y,i}$ are assumed to be normally distributed. Note that other parametrizations are also possible. More details on the choice of both functions are given in Section 2.3.

### 2.2 TOSCCA

TOSCCA expands on the standard CCA problem by reframing the estimation to a least squares problem via the Nonlinear Iterative Partial Least Squares Algorithm (NIPALS) and using soft‐thresholding that explicitly sets sparsity levels.

The TOSCCA algorithm considers the objective of CCA as a regression problem that can be solved by sequentially estimating pairs of latent variables. Each pair is estimated using the NIPALS algorithm (Wold 1966). NIPALS starts by randomly initializing one of the canonical vectors, $W_{x}^{\left(0\right)}$. Then, estimating $W_{y}$ given $W_{x}^{\left(0\right)}$ reduces to a least squares problem (Equation (1)). This process is then repeated until convergence of some tolerance measure. To deal with high‐dimensionality, TOSCCA imposes sparsity via soft‐thresholding using threshold parameters, $p_{x}$ and $q_{y}$, indicating the number of nonzero coefficients to estimate, which may take values between 1 and p or q, respectively.

TOSCCA's penalization allows us direct control on the number of nonzero weights in both $W_{x}$ and $W_{y}$, improving interpretation and speeding up the computation as we do not have to search for the penalty value that corresponds to a particular number of nonzero weights.

### 2.3 TOSCCA for Multiple Measurements

**FIGURE 2:**
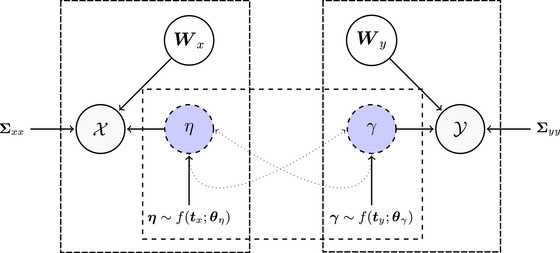
Illustration of TOSCCA‐MM. Diagram of the thresholded ordered sparse canonical correlation analysis for multiple measurements (TOSCCA‐MM) model. Observation matrices X and Y are in shaded nodes. Latent variables η and γ, in blue‐shaded dashed nodes, capture the correlation between them through sparse canonical vector $W_{x}$ and $W_{y}$. We model the longitudinal dimension in η over $t_{x}$ for each latent variable, which implicitly corrects for the correlation between measurements from the same individual. Similarly, the longitudinal dimension in γ over $t_{y}$ is modeled through $f_{t}$. See steps 3 and 8 of Algorithm 1 for more detail. We then use the estimated model to calculate predicted values for η (and similarly γ) over $t_{y}$ (and similarly $t_{x}$), steps 4 and 9. The dotted arrows indicate how these values are used to obtain the respective canonical weights, steps 5 and 10. The residual covariance matrices, $\Sigma_{xx}$ and $\Sigma_{yy}$, are made up from residual noise covariances and variation specific to each dataset.

We refer to our extended model as multiple measurements TOSCCA‐MM. The algorithm is described in pseudocode in Algorithm 1. The steps may be divided into three separate sections: one, estimating the canonical weights $W_{x}$ and $W_{y}$ in steps 5 and 10; second, we impose sparsity via soft‐thresholding in steps 6 and 11, according to the pair of thresholded parameters $\left(p_{x},q_{y}\right)$. These are further described in Section 2.2; last, we model the latent trajectories, steps 8–9 and 3–4 (Section 2.3). Note that the original TOSCCA algorithm for nonlongitudinal problems can be obtained by removing steps 3, 4, 8, and 9 of Algorithm 1. Figure 2 illustrates the concept behind TOSCCA‐MM.

We address the correlation due to multiple measurements in the latent space by modeling the time dependency in $\eta_{i}$ and $\gamma_{i}$ for all $t_{x,i}$ and $t_{y,i}$, respectively, as in Equations (2) and (3). After fitting model $f(t_{x,i};\theta_{\eta })$ over $\eta_{i}$ and $f(t_{y,i};\theta_{\gamma })$ over $\gamma_{i}$, we predict $\eta_{i}$ for $t_{y,i}$ and $\gamma_{i}$ for $t_{x,i}$ to overcome disparities between the measurements of each dataset. Given the latent variables, this approach yields conditional independence, due to the structured assumed in the longitudinal model, in which a single, dynamic latent variable is behind both observation matrices, as long as said model addressed the correlations between measurements. In doing so, we gain computational efficiency by dealing with said correlations in the low‐dimensional space. Additionally, this approach renders latent paths for all measurements of each sample. These paths represent the changing underlying trajectories for the correlated components of each dataset. After estimating our canonical weights in the lth iteration, we are left with a latent variables γ and η of n samples with at least one measurement per sample i. These latent variables describe a single latent mechanism linking both datasets, for each component k.

ALGORITHM 1 **TOSCCA‐MM**

**Table:** 

**Input**. X, Y, $W_{x}^{\left(0\right)}$, $p_{x}$, and $q_{y}$	
**Output**. $w_{x,k}^{*}$ and $w_{y,k}^{*}$	
l←1, ξ<<1, $\varepsilon =10^{6}$, $\rho^{\left(0\right)}\leftarrow 0$	
1:	**while** ε>ξ **do** ▹ Changes larger than tolerance measure ξ
2:	$\eta \leftarrow Xw_{x}^{\left(l-1\right)}$
3:	${\hat{\eta }}_{i}\leftarrow {\hat{\alpha }}_{x,i}+{\hat{\beta }}_{x}t_{x,i}$ ▹ Estimate $f_{x}$, which is a LME model with random intercepts and fixed slope
4:	${\tilde{\eta }}_{i}\leftarrow {\hat{\alpha }}_{x,i}+{\hat{\beta }}_{x}t_{y,i}$ ▹ Get predicted values for measurements in $t_{y,i}$
5:	${\tilde{w}}_{y}^{\left(l\right)}\leftarrow Y^{⊺}\tilde{\eta }$
6:	$w_{y,k}^{\left(l\right)}\leftarrow 1_{|{\tilde{w}}_{y}^{\left(l\right)}|>q_{y}}{\tilde{w}}_{y}^{\left(l\right)}-q_{y}$
7:	$\gamma_{k}\leftarrow Yw_{y,k}^{\left(l\right)}$ ▹ Standardise canonical variable for Y
8:	${\hat{\gamma }}_{i}\leftarrow {\hat{\alpha }}_{y,i}+{\hat{\beta }}_{y}t_{y,i}$ ▹ Equivalent to step 3
9:	${\tilde{\gamma }}_{i}\leftarrow {\hat{\alpha }}_{y,i}+{\hat{\beta }}_{y}t_{x,i}$ ▹ Get predicted values for measurements in $t_{x,i}$
10:	${\tilde{w}}_{x}^{\left(l\right)}\leftarrow X^{⊺}{\tilde{\gamma }}_{k}$
11:	$w_{x,k}^{\left(l\right)}\leftarrow 1_{|{\tilde{w}}_{x}^{\left(l\right)}|>p_{x}}{\tilde{w}}_{x}^{\left(l\right)}-p_{x}$
12:	$\eta_{k}\leftarrow Xw_{x,k}^{\left(l\right)}$ ▹ Standardise canonical variable for X
13:	$\rho^{\left(l\right)}\leftarrow cor\left(\eta_{k},\gamma_{k}\right)$
14:	$\varepsilon \leftarrow \rho^{\left(l\right)}-\rho^{\left(l-1\right)}$
15:	l=l+1
16:	**return** $\left(w_{x,k}^{*},w_{y,k}^{*}\right)$ ▹ Canonical weights

The choice of f(t;·) will influence the composition of the components, choosing which dynamic is represented in the model. These may yield different interpretations or representations of the underlying mechanisms in the data. Using the example in Section 2.1, in step 3 of Algorithm 1, we have $f\left(t_{x}{\hat{\theta }}_{\eta }\right)={\hat{\alpha }}_{x,i}+{\hat{\beta }}_{x}t_{x,i}$, which estimates an LME with random intercepts and fixed slope for the latent variable. We then use the estimated parameters ${\hat{\theta }}_{\eta }=\left\{{\hat{\alpha }}_{x,i},{\hat{\beta }}_{x}\right\}$ to predict the latent values over the time vector $t_{y}$ (step 4), $\tilde{\eta }=f\left(t_{y};{\hat{\theta }}_{\eta }\right)$. That is, our latent variables are expectations at times $t_{x}$ and $t_{y}$ of the longitudinal model specified for f(·). Finally, passing on these predicted latent values to estimate the alternate canonical vectors $W_{y}$, as $W_{y}^{\left(l+1\right)}={\left(Y^{T}Y\right)}^{-1}Y^{T}{\tilde{\gamma }}^{\left(l\right)}$, at each iteration l. These predicted values fill in the blanks due to sparse and irregularly observed data.

Likewise, for step 8, we have $f\left(t_{y};{\hat{\theta }}_{\gamma }\right)={\hat{\alpha }}_{y,i}+{\hat{\beta }}_{y}t_{y,i}$, where ${\hat{\theta }}_{\gamma }=\left\{{\hat{\alpha }}_{y,i},{\hat{\beta }}_{y}\right\}$. We get the predicted values over $t_{x}$ in step 9.

Through this approach, we correct for the correlated measurements ($Cov\left(x_{i,t},x_{i,v}\right)\neq 0\;\text{for}\;t\neq v,\;\left(t,v\right)\in t_{x,i}$) relying on the criss‐cross nature of the NIPALS algorithm that is useful in removing this correlation as the weights are estimated conditional on the latent variable's predicted values dealing with the correlations between measurements in the latent space, even if these are irregular and sparsely observed. Following the example of the mixed effects model above, said correlation would be contained within the random effects.. Then, assuming this one is completely described, $Cov(x_{i,t},x_{i,v}|\gamma_{i,t})=0$. Additionally we retain the selection stability properties from TOSCCA by which smaller choices of $\left(p_{x},q_{y}\right)$ yield canonical vectors that are subsets of dense alternatives when there is a signal, keeping one penalty fixed. That is, for threshold values $p_{x,1}\leq \cdots \leq p$ and some fixed $q_{y}$, both in the simulation study and the real application, $\text{supp}\left(w_{x}\left(p_{x,i}\right)\right)\subseteq \text{supp}\left(w_{x}\left(p_{x,j}\right)\right)$ if i≤j. This property is useful for interpretation of the results, as it shows selection stability of the larger contributors.

Modeling the latent variables longitudinally reveals trajectories that can be associated with the dynamics of underlying mechanisms connecting the datasets. Additionally, because the latent variables are low‐dimensional, this approach efficiently handles the correlated measurements in high‐dimensional CCA. Finally, the function f(·) is not constrained to the specifications presented in the paper, but may accommodate ones beyond the mixed effects model class, such as ARIMA models, which offers flexibility, allowing for the representation of various theories about the latent paths.

## 3 Results

### 3.1 Simulations

We simulated our data based on the probabilistic generating process described by Bach and Jordan, where we assume a single dynamic latent variable, $z_{t}$, which is equivalent to the two latent variable model we use to estimate the correlations, for they are perfectly correlated. This dynamic latent variable generates the observations at each measurement. For each measurement m=[1,10], we simulated data for n=100 samples with p=10,000 and q=200 variables in X and Y, respectively. To simulate irregular and sparsely observed measurements we artificially removed at random 20% measurements from X and 30% from Y. The same individual could have missing none, multiple or even all of their measurements. That is, for each point in time $t\in t_{x}$ and sample i∈[1,n], we generate our data following Equation (4):

(4) $$x_{i}∼N\left(z_{i}W_{x}^{T},\Psi_{x}\right),$$

where the latent variable $z_{t}$ is an n×t matrix of the latent values for all i at t and $\Psi_{x}$ is a $\bar{t}\times \bar{t}$ matrix, where $\bar{t}=\text{length}\left(t\right)$, with ones on the main diagonal and some off‐diagonal nonzeros to model time dependence. The true canonical weights are plotted in Figure 4, where we have 10 nonzero weights for each component of X and 5 for each component of Y.

**FIGURE 3:**
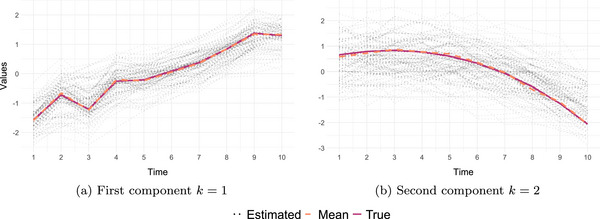
Estimated and true latent trajectories of components k=1 and k=2.

**FIGURE 4:**
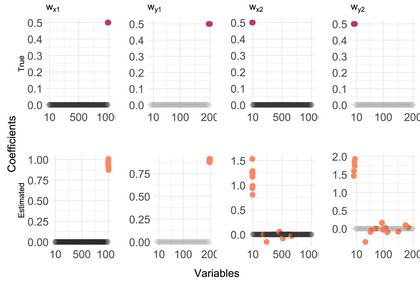
True (top) and estimated (bottom) canonical weights.

We simulated the latent trajectories for the first component as $z_{1,i}=\theta_{0}t+\sin{\left(\theta_{1}t\right)}^{t}+\varepsilon_{1,i}$ and $z_{2,i}=\theta_{2}t+{\left(1+\frac{t}{\max(t)}\right)}^{3}+\varepsilon_{2,i}$ for the second component. We followed the same scheme to generate the values for Y.

We used the TOSCCA‐MM algorithm to find $W_{x}$ and $W_{y}$ over all measurements and samples and draw the paths in the latent space. To recover the latent paths for both components (k=1 and k=2), we chose a mixed effect model $z_{k,i}∼\theta_{0,i}+P_{3}\left(t_{x,i},\theta_{1}\right)$, where $P_{3}\left(t_{x,i},\theta_{1}\right)$ is a third degree polynomial with fixed effects vector $\theta_{1}$. This model, though misspecified, is flexible enough to pick up the main trends behind the observations. The latent paths were successfully recovered, as shown in Figures 3a and 3b, where the black dotted lines represent estimated individual trajectories, the dashed blue line is the estimated average of said trajectories, and the solid red line is the true latent path. Furthermore, we estimated the optimal sparsity levels through cross‐validation for each component and found TOSCCA‐MM correctly included most relevant weights, with the exception of the canonical weights for X, $w_{x,1}$, in which, out of 10 nonzero weights and TOSCCA‐MM found 5; for the second component, we discovered the full signal with some false positives which the algorithm shrunk toward zero, reducing their contribution. We optimized the threshold parameters for each component using cross‐validation over a grid of possible pairs of $\left(p_{x},p_{y}\right)$. Hence, even when the sparsity level above the true one, the false positives remained closer to zero, giving more weight to the relevant variables.

**FIGURE A1:**
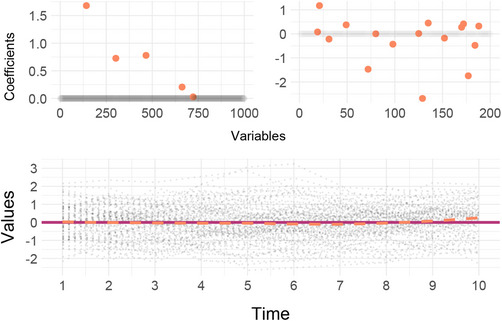
Estimated coefficients and latent path for k=3 (no signal).

Last, we calculated additional components to ensure TOSCCA‐MM returned *empty* latent paths (Figure A1, Section A.1 for k=3) and used the adjusted cumulative percentage of explained variance (Adj. CPEV) together with permutation testing (Senar et al. 2024) to assess the number of components. This analysis shows that the first two components successfully recover the true latent paths with the Adj. CPEV plateauing after the second component and the permutation testing rejecting the null hypothesis of no correlation for the first two, but not the others. The figures and details corresponding to this analysis are left to Appendix A.3.

**FIGURE 5:**
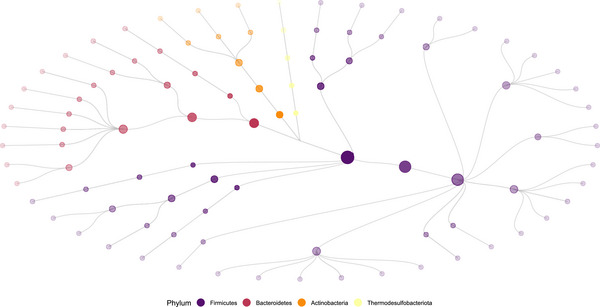
Taxonomic tree.

### 3.2 HMP Data

We used data from the HMP on gene expression measurements and gut microbiome signatures from 84 healthy and prediabetic individuals (Zhou et al. 2019) and looked into the gut–gene associations that may differ between IS and IR individuals.

The gut microbiome in our data is divided into four big groups: Firmicutes, Bacteroidetes, Actinobacteria, and Thermodesulfobacteriota. The first two make up 90% of humans gut microbiota. These groups are organized into five hierarchical aggregation levels: genus, family, order, class, and phylum, with phylum representing the highest level of aggregation. We show in Figure 5 these groups by color and the levels by shades. That is, the nodes on the outer side with the lightest shade represent OTUs at the genus level. Each step into the center inward aggregates the previous levels.

#### 3.2.1 Gut–Gene Associations and Prediabetes

Prediabetes is a condition arising from insufficient production of insulin in the pancreas to maintain normal blood glucose levels. It is considered a precursor to diabetes, as individuals with prediabetes have elevated blood glucose levels, though below those of diabetic patients. Most people with prediabetes experience IR and many with IR eventually develop prediabetes.

In the past decade, studies have widened the scope of mechanisms involved in the development of (pre‐)diabetes and diabetes linking inflammatory responses and other metabolic responses and gut microbiome to the development of IR. The gut microbiome is a balanced ecosystem unique to each individual affecting multiple layers of human health. Among those layers, the composition of the gut microbiota plays an important role in the continuous development of the immune system. Alterations in said composition are connected to IR (Semo et al. 2024; Takeuchi et al. 2023; Wu et al. 2020). Particularly, the abundance of some microbes linked to inflammation, such as *Prevotella* and some groups of *Clostridiales* and *Actinobacteria*, has been used to predict risk of prediabetes (Alvarez‐Silva et al. 2021; Pinna et al. 2021). These alterations may be triggered by health disruptions as healthy individuals have different responses compared to their IS counterparts (Zhou et al. 2019).

A host of genetic factors are involved in the makeup of the gut microbiota as well as influence its alterations(Nochols and Davenport 2020). Genome‐wide association studies (GWAS) have found that genetic variants could alter gene expression patterns that, in turn, may have an effect on the intensity of the immune response (Diedisheim et al. 2020; Nochols and Davenport 2020). Additionally, IR patients show differences in their gene expression patterns related to key enzymes responsible for processing glucose (Patti 2004).

**FIGURE 6:**
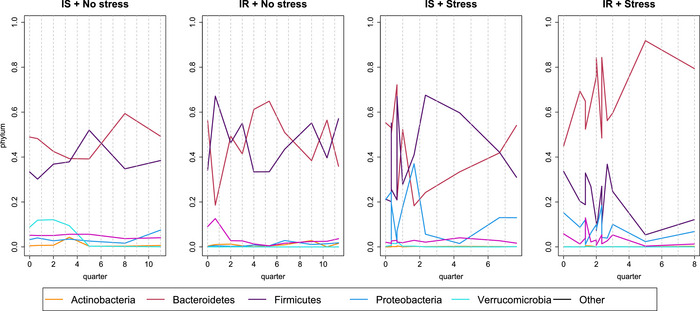
OTUs measurements at phylum level for four patients.

We observed this pattern in our data looking at the dynamics of the OTUs at the phylum level for patients who were IS, IR, and with or without a negative health event during the study, for which data are available. We referred to these visits as *stress* visits, as opposed to healthy, regular visits. Figure 6 shows examples for all four scenarios. Patients with no additional *stress* to their health had more stable OTU values than those who did experienced it, while IS and IR patients appeared to be affected by these differently.

#### 3.2.2 Results from TOSCCA‐MM Analysis of HMP Data

We used measurements on over 10,000 genes and 21 OTUs at the family level.^1^ Each individual had between 1 and 80 measurements approximately every 3 months. As mentioned in Section 1, these were irregularly and sparsely observed. That is, visits were not recorded at all times for every individual, or there were multiple visits within those 3 months. The latter occurred when an important event in the patient's health was detected (usually via self‐report). The most common of these events were falling sick (and taking medication) and weight changes. These were all categorized as *stress* visits. We kept the first 19 measurements for each view, after which data were available on very few patients.

**FIGURE 7:**
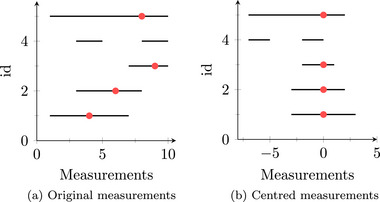
Original and centered measurements around an event (red dot).

We rearranged the longitudinal dimension by summarizing visits into 3‐month intervals centered around the first *stress* visit (Figure 7). Measurements from patients without a reported *stress* visit were shifted to the left (Figure 7a). These changes turned our multiple measurements into comparable time series among all individuals and allowed us to observe the latent mechanisms around health events that are known to follow different mechanisms between healthy and glucose‐dysregulated people. Last, we divided the observations into two groups, IS and IR, and performed the analysis on each group.

We used the TOSCCA‐MM algorithm to find the canonical vectors and fit their latent trajectories. We found the optimal threshold parameters $p_{\alpha }$ and $q_{\beta }$ via cross‐validation over a grid of possible combinations. Because we centered our time series along a particular event, we chose a change‐point mixed‐effect model with random intercepts and a polynomial slope (Equation (5)). Different choices of parametrization for Equation (5) would, of course, render different compositions, or change the order in which the components appeared.^2^ The specification in equation above was general enough to find trend, inflection, or/and change points with random intercepts.

(5) $$\begin{array}{c} \gamma_{i,t}=a_{0,i}+\sum_{r}^{3}a_{r}t^{r}+\beta t1_{t>s}+\varepsilon_{i,t}. \end{array}$$

**FIGURE 8:**
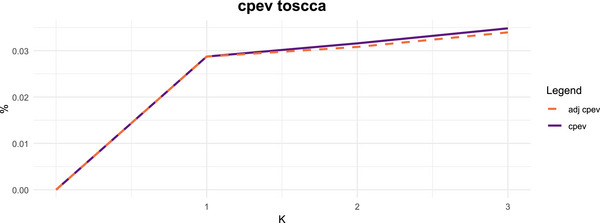
CPEV for the microbiome datasets.

Although permutation testing is available, the primary objective in this analysis is not formal inference on the estimated associations but rather to explore potential paths that bring insights into the underlying mechanisms linking the gut microbiome and gene expression. Given the data structure at hand, there may be many components that are deemed significant under permutation, making such testing less informative for component selection than in settings with a clear aim on inference. We therefore emphasize that the choice between inferential and exploratory analysis should be guided by the research objective. When inference is the goal, it is appropriate to perform permutation testing for some given sparsity levels. In contrast, for exploratory aims, summary measures such as the adjusted cumulative proportion of explained variance (CPEV) offer a more useful criterion for interpreting and selecting components. Figure 8 displays the latter, based on which we would assess there are more components to explore. For brevity, we will focus on the first two.

**FIGURE 9:**
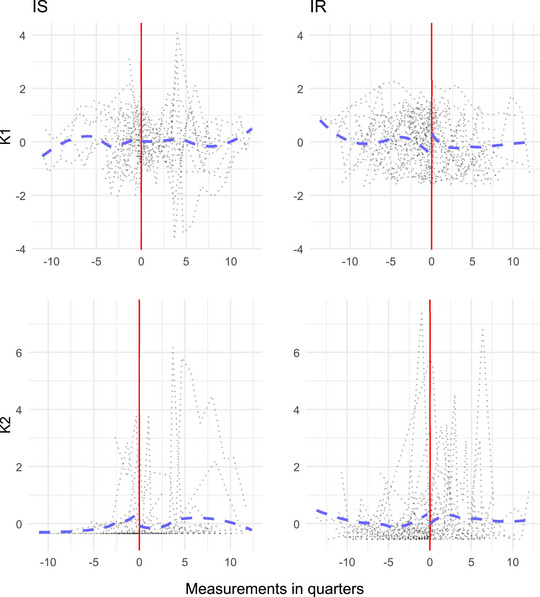
Estimated latent paths for component k=1 and k=2.

The latent trajectories for the first two components are plotted in Figure 9. The first component was made up from the most abundant OTUS for both groups and appeared to show similar latent trajectories representing general links between the gut microbiota and the host gene expressions. Hence, picking up on the general conditions of the gut microbiome for both groups, with their latent trajectories selecting the same OTUs. Additionally, the path for the IR group showed a disturbance at the moment of the health event compared to the healthy group. This could lead back to IR patients experiencing grater disruptions than their healthy counterparts in such episodes. On the other hand, the second component was composed of different OTUs for each group. The latent mechanism for the IS group consisted of *Prevotellaceae*, linked to inflammatory events, generally responding to altered immune and gut environments an insulin regulation. For the IR group, *Clostridiales* and *Coriobacteriaceae* were the relevant OTUs. The latter is associated to insulin sensitivity mechanism through its production of *butyric acid*. *Coriobacteriaceae* have been linked to benefits in insulin sensitivity in the prevention of the development of type 2 diabetes. The second component appeared to put forward differences between groups with the IR group giving relevance to OTUs important for insulin regulation.

The above may indicate that the first component represented the trivial association between genetics and gut composition; while the second indeed described differences between IS and IR trajectories under stress. Thus, suggesting relevant latent dynamics are different between these groups.

With respect genetic contributors to each mechanism, we observed a similar pattern in the first component. The main contributors were shared between both IS and IR individuals consisting of around 200 estimated nonzero canonical weights. For the second component, however, the estimated nonzero weights were not the same. This once more indicated differences between the groups on the dynamics which we previously linked to the *stress* events, on the genetic level.

Whether these differences in component composition stem from reactions associated with their IS or IR status, or from tendencies toward distinct health disruptions within each group, falls outside the scope of this paper.

## 4 Conclusions

In this paper, we introduced a novel method, TOSCCA‐MM, for building latent trajectories linking two datasets two high‐dimensional datasets over time. Our approach captures the evolving dynamics of associations between variables by keeping the sparse canonical weights fixed across all measurements. TOSCCA‐MM contrasts with existing methods that typically focus on correcting the correlations between measurements or estimating canonical weights at each point in time. These methods struggle with high dimensional, sparse and irregularly observed data or fail to exploit the longitudinal aspect of the repeated measurements. By fixing the canonical weights, TOSCCA‐MM prioritizes uncovering the underlying processes driving the relationships between variables as they change together over time, rather than merely describing static correlations at each measurement. This enables us to draw latent trajectories of the most relevant features for each component, providing deeper insights into the hidden, time‐dependent links between observation matrices.

TOSCCA‐MM identifies sets of variables that are consistently highly correlated across measurements, and models how these correlations evolve over time in the latent space. This approach highlights key contributors to the latent dynamics and enables modeling of more complex patterns, such as lagged effects or variables that switch on and off over time. These features are directly built into TOSCCA‐MM, making it more robust and interpretable compared to existing methods, which often struggle with the dimensionality of the data and the complexity of longitudinal relationships.

TOSCCA‐MM is particularly well suited for handling high‐dimensional data with irregular and sparsely observed data. The implementation of TOSCCA‐MM is available as open‐source code on GitHub (https://github.com/nuria‐sv/toscca‐mm).

## Conflicts of Interest

None declared.

## Funding

None declared.

## Open Research Badges

This article has earned an Open Data badge for making publicly available the digitally‐shareable data necessary to reproduce the reported results. The data is available in the Supporting Information section.

This article has earned an open data badge “**Reproducible Research**” for making publicly available the code necessary to reproduce the reported results. The results reported in this article could fully be reproduced.

## Supporting information

Supporting Information

## Figures and Tables

**FIGURE A2:**
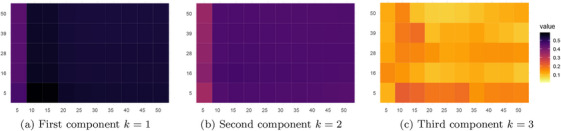
Heatmaps from cross‐validation results. Options for $p_{x}$ are ordered in the x‐axis and $p_{y}$ in the y‐axis.

**FIGURE A3:**
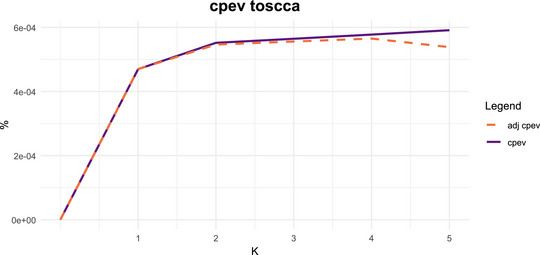
CPEV and adjusted CPEV for K=5.

**FIGURE A4:**
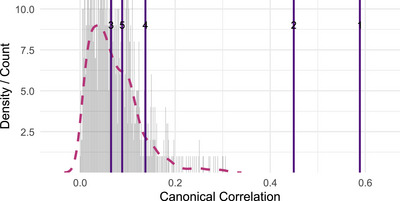
Permutation testing. Empirical density under the null of no correlation is in purple, and estimated canonical correlations for the simulated data are the vertical numbered lines in dark blue.

**FIGURE A5:**
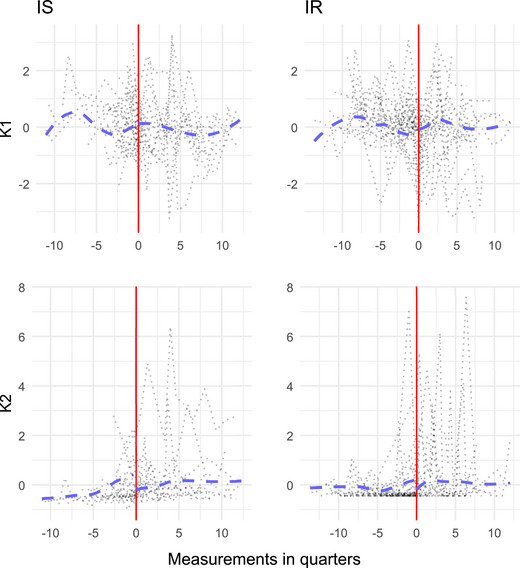
Estimated latent paths for k=1 and k=2 by group at the genus level.

**FIGURE A6:**
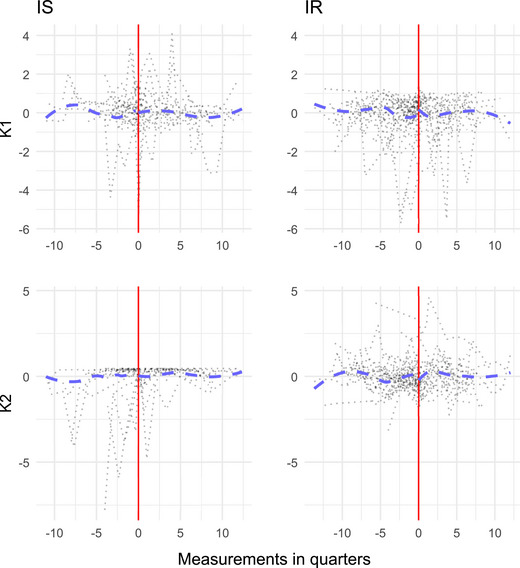
Estimated latent paths for k=1 and k=2 by group at the order level.

**FIGURE A7:**
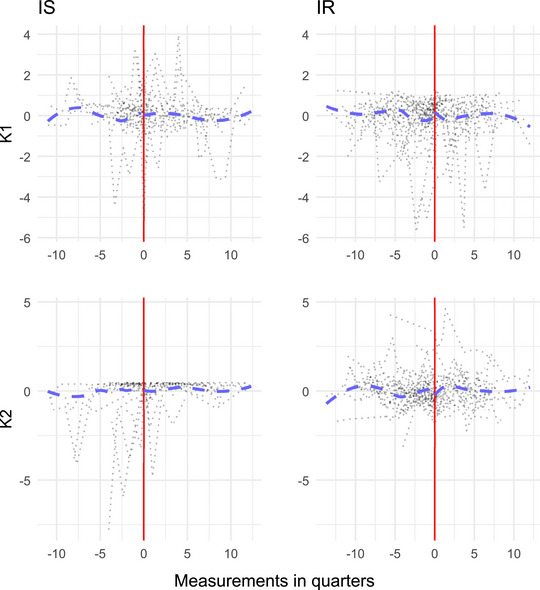
Estimated latent paths for k=1 and k=2 by group at the class level.

**FIGURE A8:**
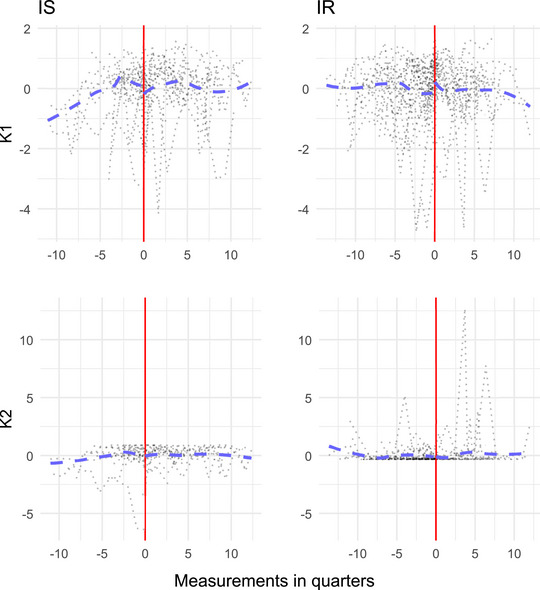
Estimated latent paths for k=1 and k=2 by group at the phylum level.

## Data Availability

The data that support the findings of this study are available in Stanford iPOP site at http://hmp2‐data.stanford.edu/. These data were derived from the following resources available in the public domain: ‐ Zhou, W., Sailani, M. R., Contrepois, K., et al. Longitudina, https://www.nature.com/articles/s41586‐019‐1236‐x#data‐availability

## Endnotes

1 Results for the other taxonomic levels are left to Appendix A.4 for brevity.

2 For this case, we compared alternative specifications for the latent trajectories and observed virtually equivalent components with minor changes at some of the taxonomic levels.

## Appendix A

### A.1 Simulations

Figure A1 displays the estimated coefficients and latent path for k=3 for which there is no signal. TOSCCA‐mm selected some coefficients to be nonzero, most of them with values close to zero, with some extreme exceptions. The latent variable is correctly set to zero for all possible measurements.

### A.2 Cross‐Validation for Optimal Sparsity Levels

Figure A2.

### A.3 Choosing the Number of Components (K)

The dimensions of the canonical weights are chosen via the adjusted cumulative percentage of explained variance (Adj. CPEV) together with the results from the permutation testing.

The original CPEV is defined as $tr(\eta_{k}^{⊺}\eta /tr\left(X_{s}^{⊺}X_{s}\right)$, for some observation matrix X at measurement s with corresponding latent variable $\eta_{k}$. This measure may be inadequate for high dimensions because orthogonality of the canonical vectors can no longer be guaranteed, new components do not necessarily contain new information, and hence latent variables may be correlated. We propose the following alternative measure to adjust for repeated information for k=2.⋯,K:

(A1)
$$\begin{array}{cc} & \text{Adj.}\;CPEV\left(\eta_{k}\right)=CPEV\left(\eta_{k}\right)\cdot \prod_{i<k}(1-|cor\left(\eta_{i},\eta_{k}\right)\left|\right), \end{array}$$

As a general rule of thumb, the choice of dimensions, or number of components, is to cut off once the CPEV with respect to the number of components plateaus. The results of the CPEV and Adjusted CPEV are displayed in Figure A3. The largest increases of explained variance are in the first two components and the Adjusted CPEV plateaus at the third one.

Additionally, we use permutation testing to further analyze the relevance of the estimated components. In order to permute multiple measurement matrices, we fixed the id and measurement columns and permuted the rows of the features of X and estimated the correlations under the null for 1000 draws. The results are displayed in Figure A4, where we confirm the first two components leading to correlations significantly different (*p*‐values: 0.000,0.000,0.446,0.099,0.511) as those coming from the null distribution under no correlation.

One important aspect of permutation testing for multiple components is that the null distribution of canonical correlations must be estimated using the same sparsity levels than the correlation being tested, as otherwise the correlations may arbitrarily change. Null distributions contingent on sparsity levels may lead to incorrect assessment of the estimated canonical correlations. This is another issue of standard penalties, such as elastic net or lasso, as the same penalty across permutation may not return the same sparsity level, hence producing invalid null distributions.

In summary, we estimated five components for which the adjusted CPEV plateaus after the second component, and after performing permutation testing, we can conclude that the two true latent variables are recovered.

### A.4 HMP Data

Below we provide a quick summary of the estimated canonical vectors for the OTU's at the remaining levels: from most disaggregated (genus) to most aggregated (phylum). Different aggregation levels may put forward different dynamics; hence, we did not expect all estimates to perfectly align. In fact, some middle aggregation levels may mask smaller variations from different bacteria that may be visible at lower levels. On the other hand, higher levels may pick up on global effects for the different mayor microbiome groups.

For all levels, including at the family level in the main text, the first component (k=1) highlighted general contributions and dynamics in both IS and IR patients. That is, the canonical vectors for both groups had virtually identical contributors for the first latent path. For that, in this section, we focused on the second component (k=2) for which groups exhibited clear differences.

#### A.4.1 Genus

The estimated latent paths at genus level are displayed in Figure A5. The canonical weights at the genus level for k=2 are not dissimilar to those discuss in the main text. The second component highlights OTU's linked to insulin sensitivity and anti‐inflammatory systems, for the IS group. For the IR group, the only OTU for component k=2 *Clostridiales Incertae Sedis XIII*.

#### A.4.2 Order

The latent paths at order level are displayed in Figure A6. The estimated canonical weights for k=2 are somewhat inconclusive at this level. Second component is composed exclusively of *Deltaproteobacteria*, with negative weight, for the IS group. This bacteria has been found to be more prevalent in prediabetic patients (Chen et al. 2021). However, it has been suspected to have some anti‐inflammatory benefits (Xia et al. 2021). On the other hand, the IR group is for once more diversified, including most of the bacteria groups in the composition of the second latent variable (10 out of 12). These differences with the previous levels might be explained by the aggregation level combining changes that were previously disaggregated.

#### A.4.3 Class

The estimated latent paths at the phylum level are displayed in Figure A7. At this level, the analysis is equivalent to the one at order level.

#### A.4.4 Phylum

The estimated latent paths at the phylum level are displayed in Figure A8. At this level, we observe six OTU's. Again, the *Bactiroidetes* and *Firmicutes* groups represent around 90% of gut microbiota in a healthy individual. The *Actinobacteria* and *Proteobacteria* groups are often the ones undergoing larger changes in individuals with disorders affecting the microbiome balance. The estimated canonical weights for k=2 gave relevance to the *Actinobateria* OTU that has been consistently linked to IR. For the IS patients, we are unable to dive in further as the chosen microbe group was unclassified in the dataset. These results are in line with those presented in the main text.
