# Supplementary material for: Sparse Canonical Correlation Analysis for Multiple Measurements With Latent Trajectories
Source: Biom J. 2025 Oct 30;67(6):e70090. doi: 10.1002/bimj.70090 (PMC12573309; doi:10.1002/bimj.70090)
Supplement: Supplementary file 1 — Supporting Information [file BIMJ-67-e70090-s001.zip › tosccammCode_senar/readmeKnit.pdf]

tosccamm

status finished | R passing | license MIT

Installation

NOT READY

You can install the development version of tosccamm like so:

```
devtools::install_github("nuria-sv/toscca")    # dependencies
devtools::install_github("nuria-sv/tosccamm")
```

TOSCCA-MM

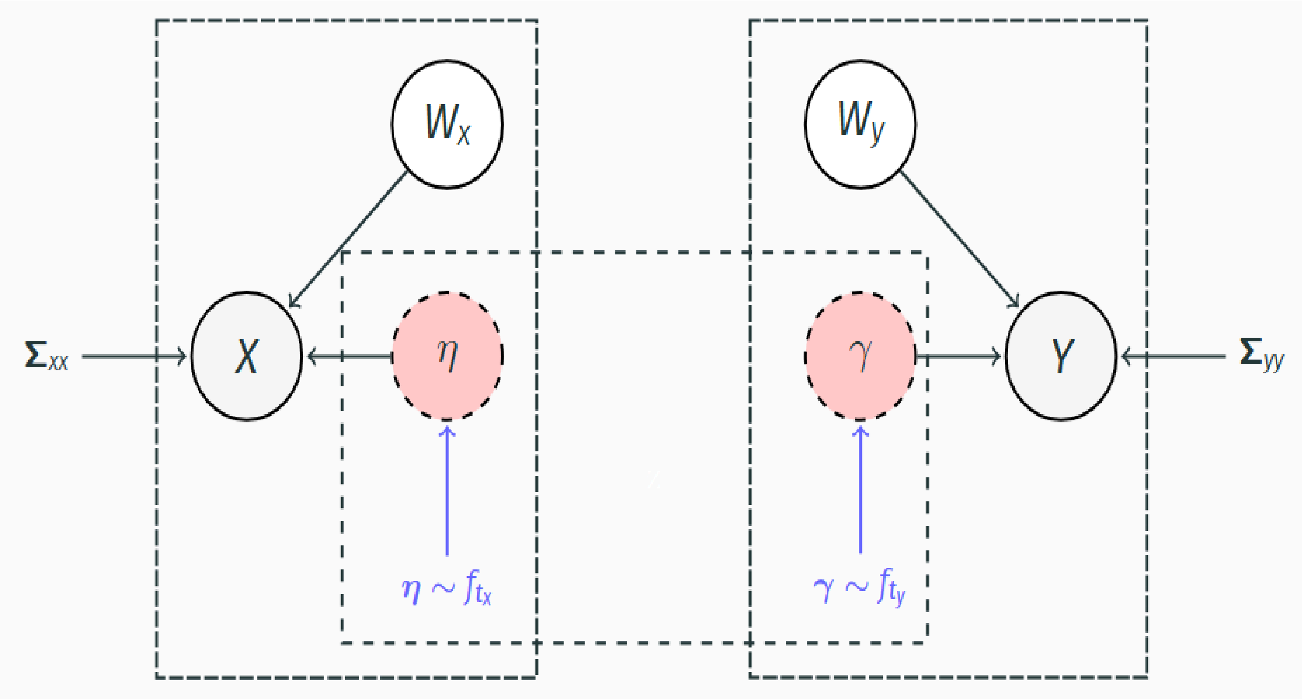

TOSCCA-MM is a novel extension of sparse CCA that incorporates time dynamics at the latent variable level through longitudinal models, such as autoregressive models or linear mixed effect models. This approach addresses the correlation of repeated measurements while drawing latent paths, for each component. To aid interpretability and computational efficiency, we implement an ℓ_0 penalty to enforce fixed sparsity levels. We estimate these trajectories fitting longitudinal models to the low-dimensional latent variables, (i.e.: linear

mixed effects model). By leveraging the clustered structure of high-dimensional datasets, we are able to explore the shared longitudinal latent mechanisms. The sparse canonical weights, yield interpretable outcomes on variable contribution to the estimated correlated trajectories. Furthermore, modelling time in the latent space significantly reduces computational burden.

Session details

R version 4.4.3 (2025-02-28 ucrt) Platform: x86_64-w64-mingw32/x64 Running under: Windows 11 x64 (build 26100)

Matrix products: default

locale: [1] LC_COLLATE=English_United States.utf8 LC_CTYPE=English_United States.utf8
[3] LC_MONETARY=English_United States.utf8 LC_NUMERIC=C
[5] LC_TIME=English_United States.utf8

time zone: Europe/Amsterdam tzcode source: internal

attached base packages: [1] stats graphics grDevices utils datasets methods base

loaded via a namespace (and not attached): [1] compiler_4.4.3 fastmap_1.2.0 cli_3.6.3 htmltools_0.5.8.1 tools_4.4.3
[6] rstudioapi_0.17.1 yaml_2.3.10 rmarkdown_2.29 knitr_1.49 xfun_0.50
[11] digest_0.6.37 rlang_1.1.5 evaluate_1.0.3

Example

This is a basic example over simulated data of TOSCCA-MM

```
# library(tosccamm)
source("R/tosccam_permut.R")
source("R/tosccamm_core.R")
source("R/tosccamm_folds.R")
source("R/toscca_helpers.R")
source("R/general_functions.R")
# for plots
library(grid)
library(ggplot2)
library(gridExtra)
```

```
library(viridis)
#> Loading required package: viridisLite
```

Estimate the canonical weights and latent paths for K components.

```
res_k = list()

X.temp = XX2
Y.temp = YY2
for (k in 1:5) {
  if(k > 1) {
    # residualise for subsequent components
    X.temp = data.frame(X.temp[,c(1,2)],toscca::residualisation(as.matrix(X.temp[,-c(
    Y.temp = data.frame(Y.temp[,c(1,2)],toscca::residualisation(as.matrix(Y.temp[,-c(

    nz_a_gen = as.numeric(table(res_k[[k-1]]$alpha != 0)[2])
    nz_b_gen = as.numeric(table(res_k[[k-1]]$beta != 0)[2])
  }

  res_k[[k]] <- tosccamm(X.temp, Y.temp, folds = 2,
                                          nonzero_a = nonz_a, nonzero_b = nonz_b,
                                          model = "lme", lmeformula = " ~ 0 + poly(

}
#>  Common convergence error: 0 & Iterations: 5
#> Warning in any(m): coercing argument of type 'double' to logical
#>  Common convergence error: 0 & Iterations: 5
#> Warning in any(m): coercing argument of type 'double' to logical
#>
#> k-fold cv max. cancor
#>             0.5885827
#>
#>  ........................................
#>  # nonzero A: 10
#>  # nonzero B: 5
#>  ........................................
#>  Common convergence error: 0 & Iterations: 17
#> Warning in any(m): coercing argument of type 'double' to logical
#>  Common convergence error: 0 & Iterations: 13
#> Warning in any(m): coercing argument of type 'double' to logical
#>
#> k-fold cv max. cancor
#>             0.4677066
#>
#>  ........................................
#>  # nonzero A: 15
#>  # nonzero B: 16
#>  ........................................
```

```
#>  Common convergence error: 0.00881 & Iterations: 21
#> Warning in any(m): coercing argument of type 'double' to logical
#>  Common convergence error: 0.02011 & Iterations: 21
#> Warning in any(m): coercing argument of type 'double' to logical
#>
#> k-fold cv max. cancor
#>             0.2185483
#>
#>  ........................................
#>  # nonzero A: 10
#>  # nonzero B: 5
#>  ........................................
#>  Common convergence error: 0.01257 & Iterations: 21
#> Warning in any(m): coercing argument of type 'double' to logical
#>  Common convergence error: 0.02866 & Iterations: 21
#> Warning in any(m): coercing argument of type 'double' to logical
#>
#> k-fold cv max. cancor
#>             0.1567649
#>
#>  ........................................
#>  # nonzero A: 30
#>  # nonzero B: 39
#>  ........................................
#>  Common convergence error: 0.01773 & Iterations: 21
#> Warning in any(m): coercing argument of type 'double' to logical
#>  Common convergence error: 0.02479 & Iterations: 21
#> Warning in any(m): coercing argument of type 'double' to logical
#>
#> k-fold cv max. cancor
#>             0.2434016
#>
#>  ........................................
#>  # nonzero A: 50
#>  # nonzero B: 16
#>  ........................................
```

Results

Latent paths for $\mathrm{k} = 1$ and $\mathrm{k} = 2$

Figures 3.a and 3.b in manuscript.

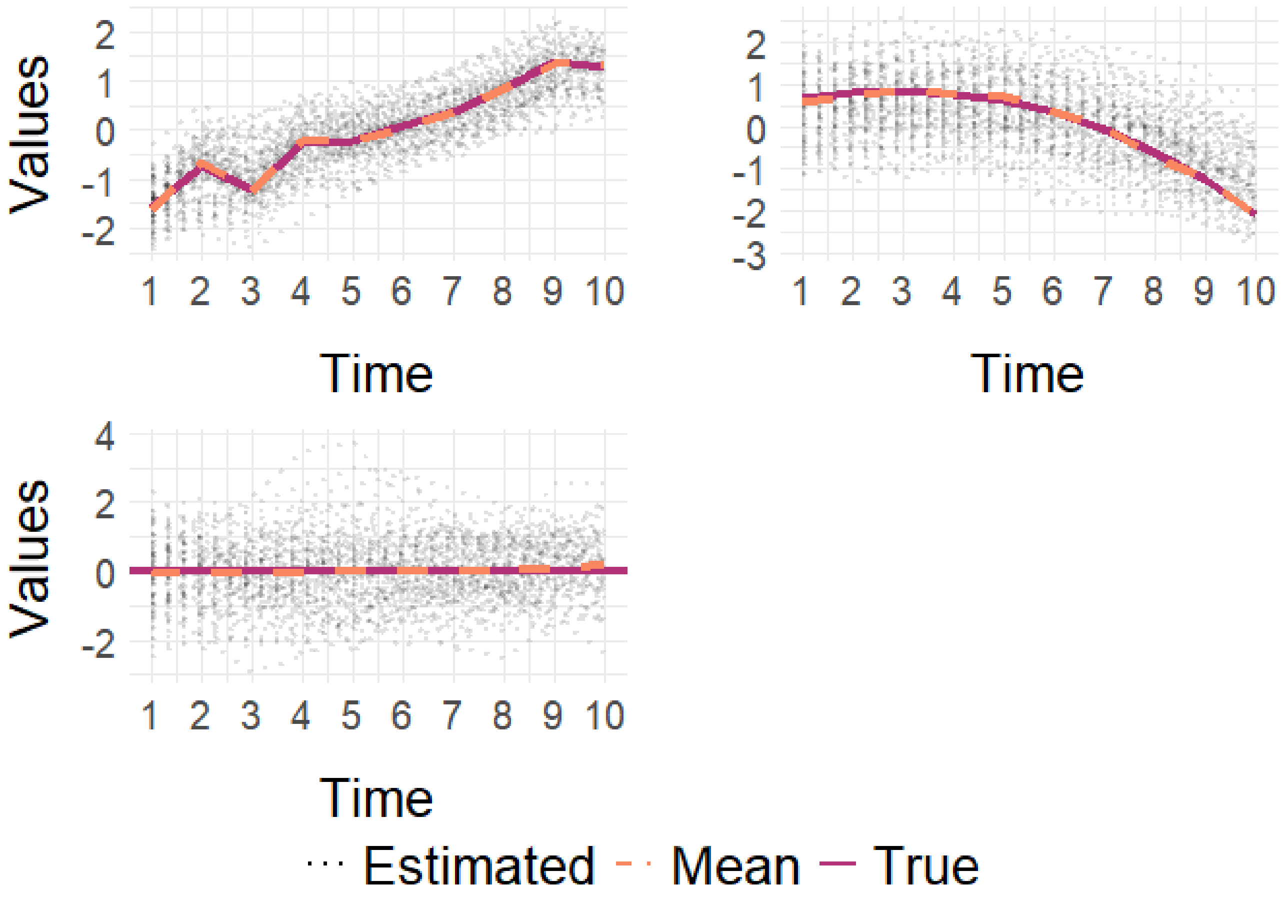

Canonical weights for $k = 1$ and $k = 2$

Figure 4 in manuscript.

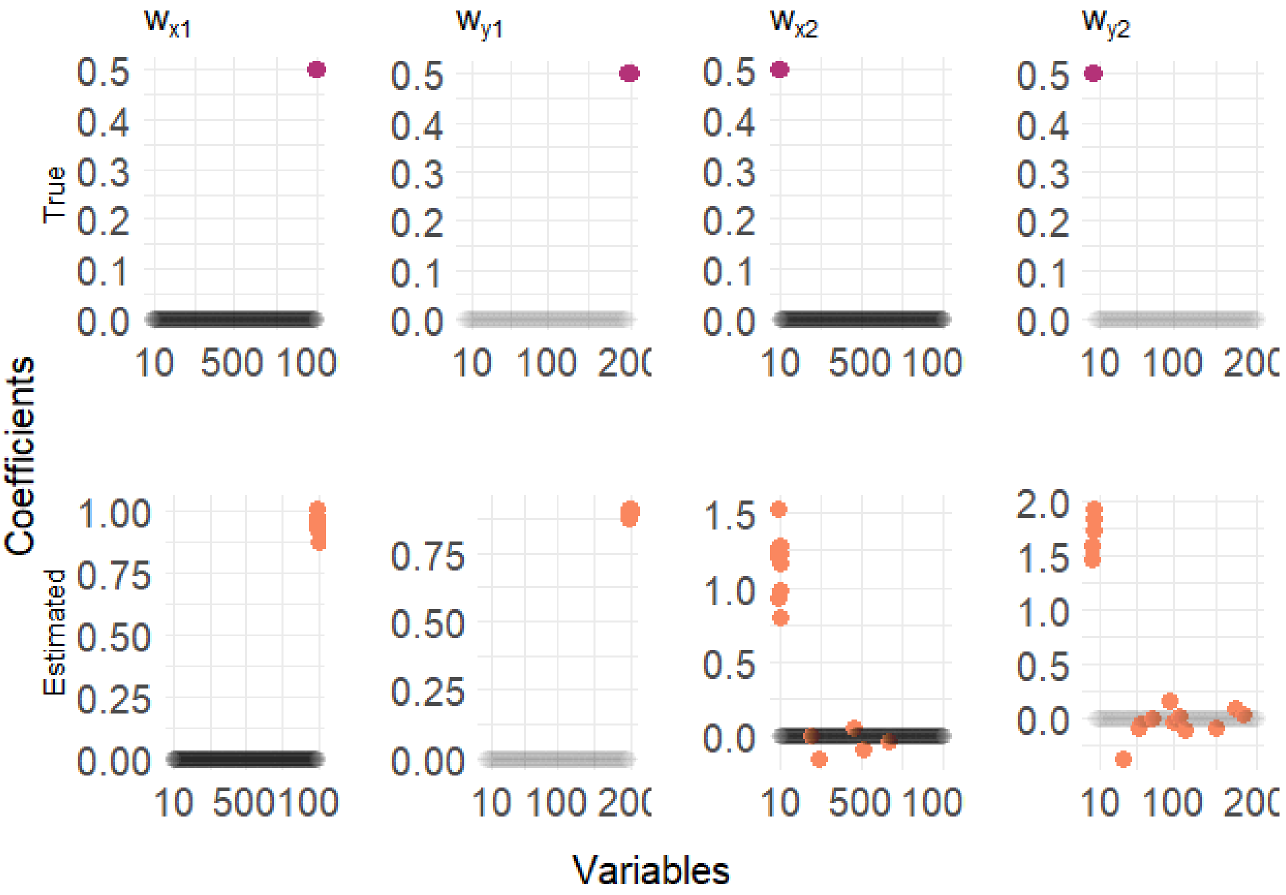

Latent path and canonical weights for $k = 3$, noise

Figure 10 in manuscript.

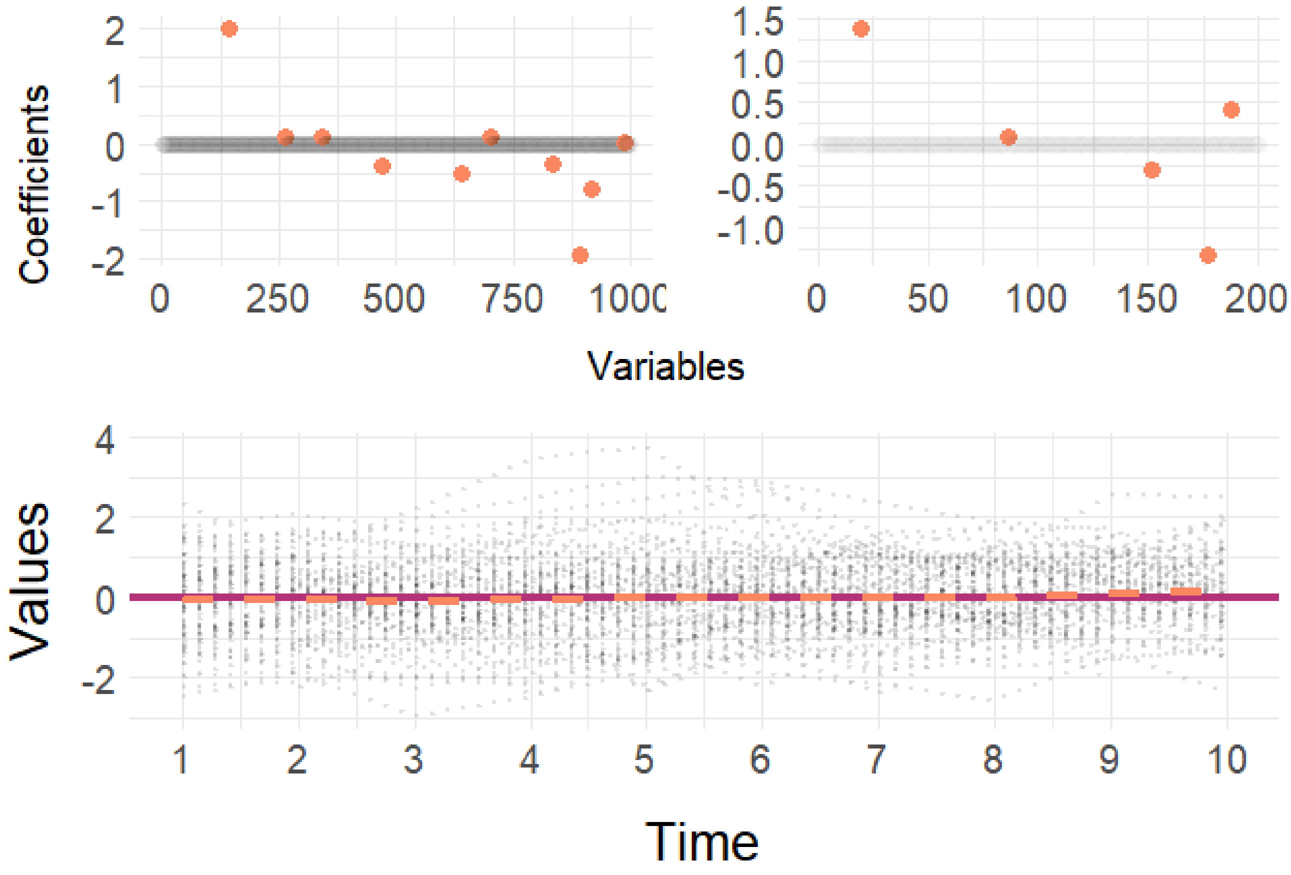

Figure 11 in manuscript.

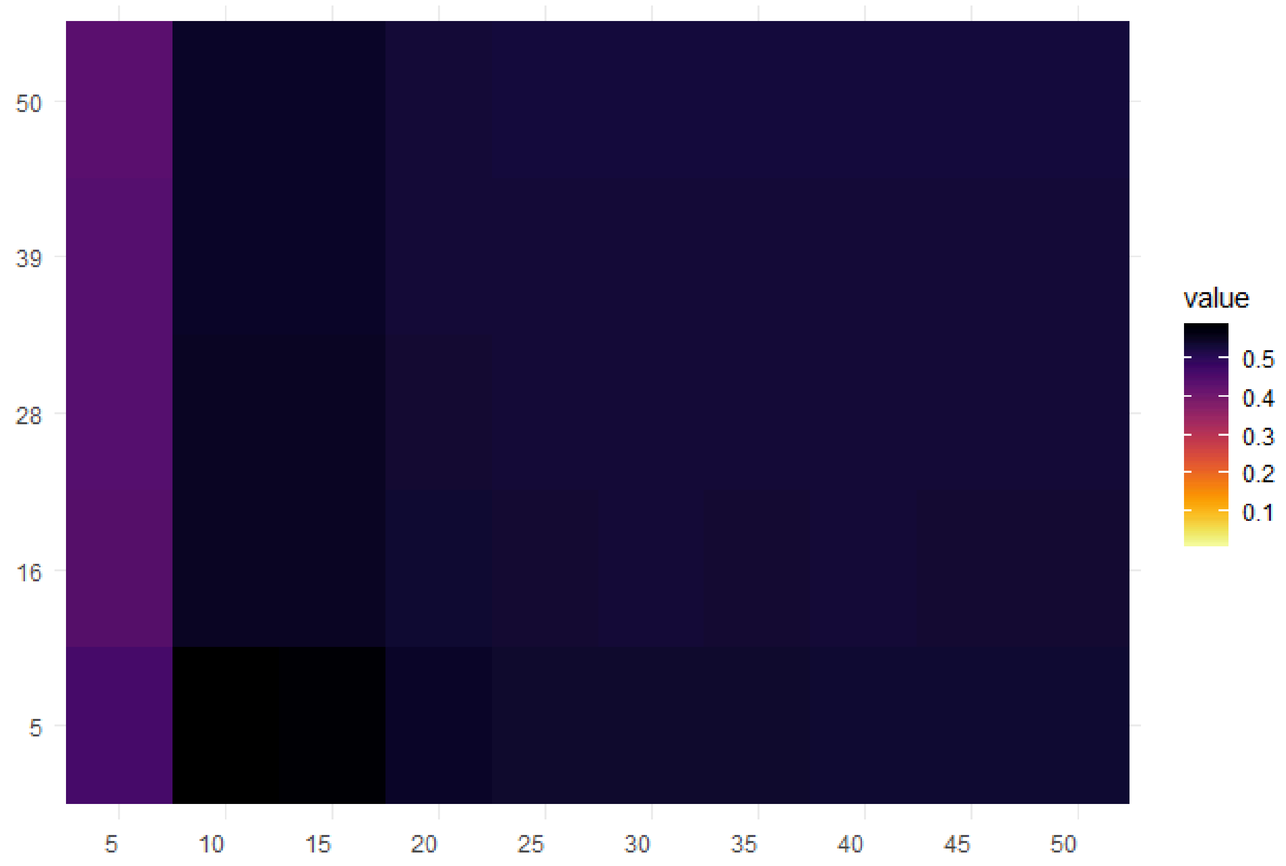

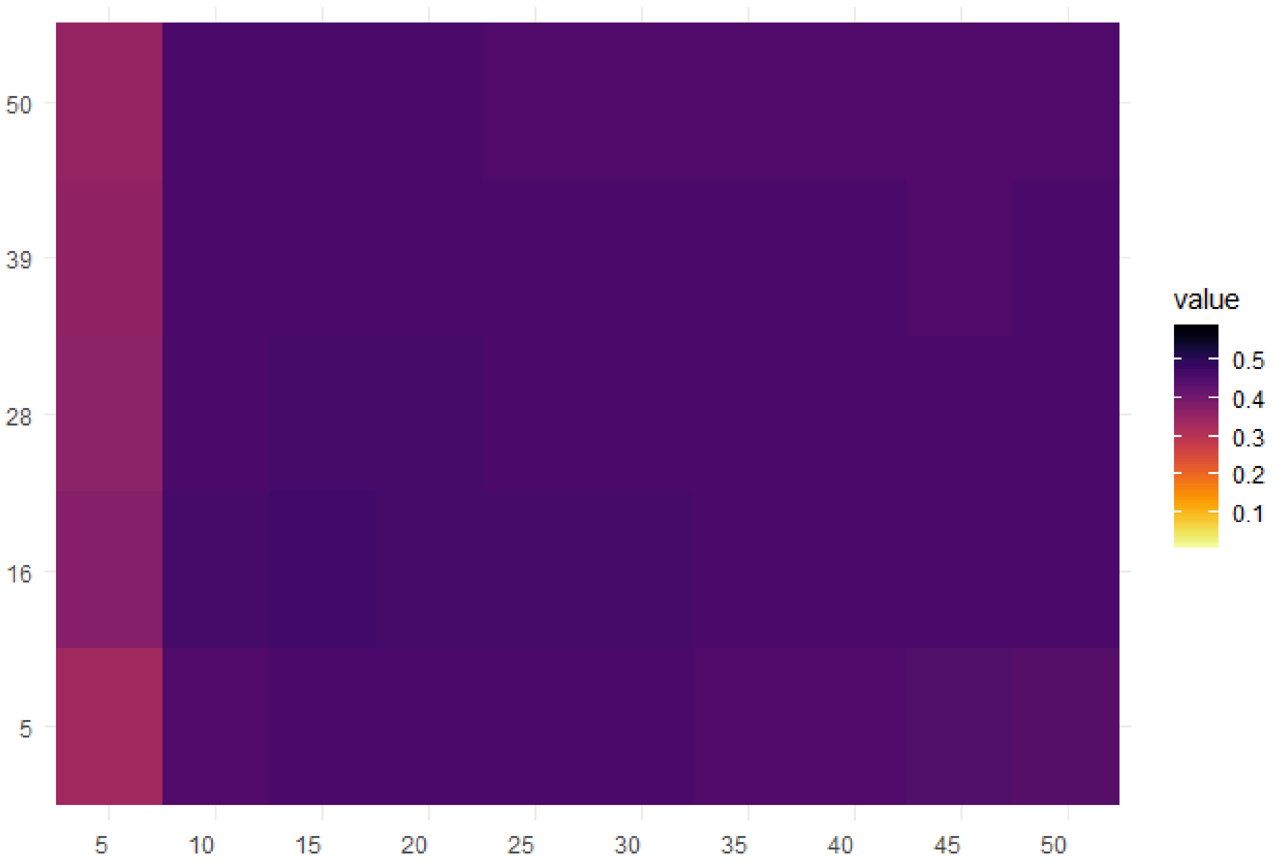
50
39
28
16
5
5
10
15
20
25
30
35
40
45
50
value
0.5
0.4
0.3
0.2
0.1

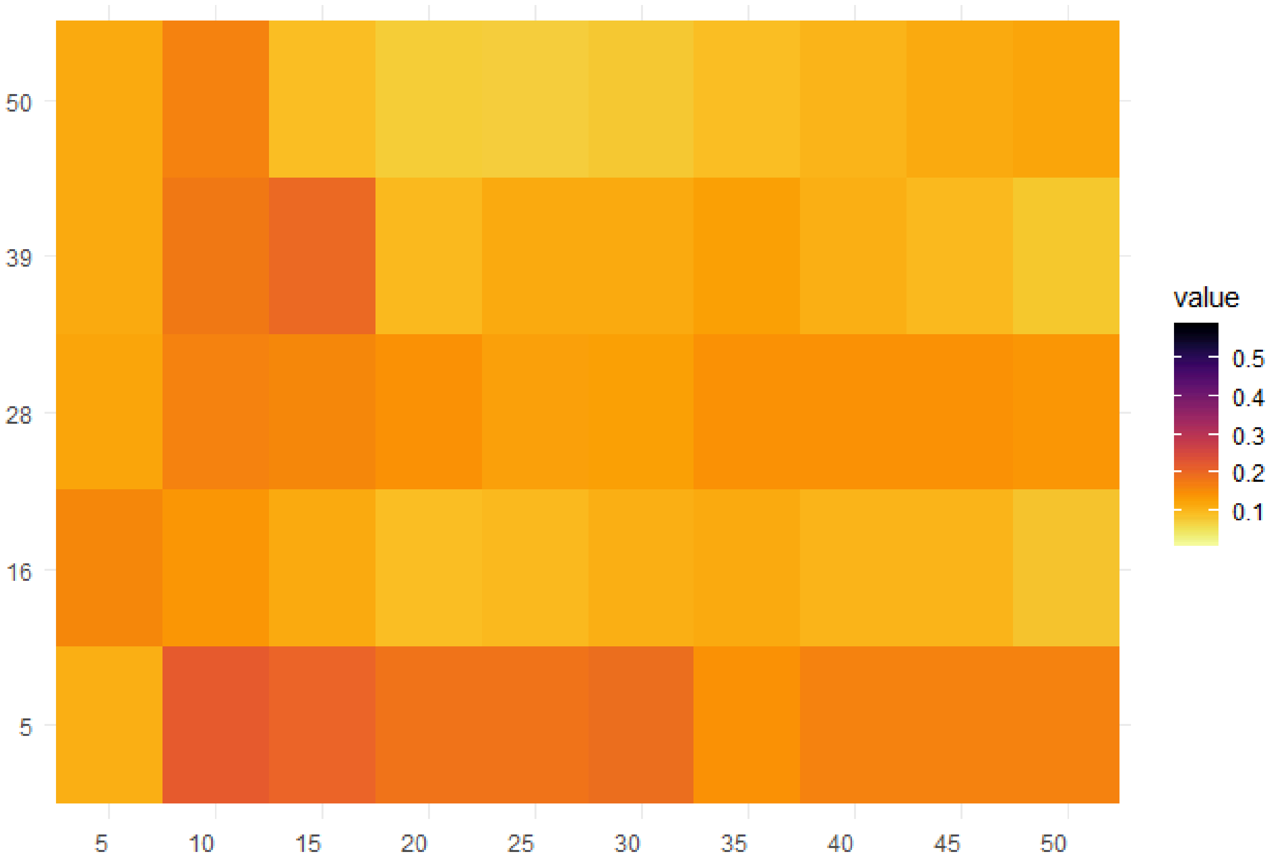
50
39
28
16
5
5
10
15
20
25
30
35
40
45
50
value
0.5
0.4
0.3
0.2
0.1

Figure 13 in manuscript.

```
#>  Common convergence error: 0 & Iterations: 5
#> Warning in any(m): coercing argument of type 'double' to logical
#>  Common convergence error: 0 & Iterations: 5
#> Warning in any(m): coercing argument of type 'double' to logical
#>
#> k-fold cv max. cancor
#>             0.5885827
#>
#>  .......................................
#>  # nonzero A: 10
#>  # nonzero B: 5
#>  .......................................
#>  Common convergence error: 0 & Iterations: 7
#> Warning in any(m): coercing argument of type 'double' to logical
#>  Common convergence error: 0 & Iterations: 8
#> Warning in any(m): coercing argument of type 'double' to logical
#>
#> k-fold cv max. cancor
#>             0.4494961
#>
#>  .......................................
#>  # nonzero A: 10
#>  # nonzero B: 5
#>  .......................................
#>  Common convergence error: 0.05982 & Iterations: 21
#> Warning in any(m): coercing argument of type 'double' to logical
#>  Common convergence error: 0 & Iterations: 21
#> Warning in any(m): coercing argument of type 'double' to logical
#>
#> k-fold cv max. cancor
#>            0.06794261
#>
#>  .......................................
#>  # nonzero A: 10
#>  # nonzero B: 5
#>  .......................................
#>  Common convergence error: 0.02629 & Iterations: 21
#> Warning in any(m): coercing argument of type 'double' to logical
#>  Common convergence error: 0 & Iterations: 21
#> Warning in any(m): coercing argument of type 'double' to logical
#>
#> k-fold cv max. cancor
#>             0.1479751
#>
#>  .......................................
#>  # nonzero A: 10
```

```
#>  # nonzero B: 5
#>  .......................................
#>  Common convergence error: 1e-05 & Iterations: 21
#> Warning in any(m): coercing argument of type 'double' to logical
#>  Common convergence error: 0 & Iterations: 17
#> Warning in any(m): coercing argument of type 'double' to logical
#>
#> k-fold cv max. cancor
#>           0.06081026
#>
#>  .......................................
#>  # nonzero A: 10
#>  # nonzero B: 5
#>  .......................................
#> Loading required package: iterators
#> Loading required package: parallel
#> Warning in e$fun(obj, substitute(ex), parent.frame(), e$data): already
#> exporting variable(s): K
#> Warning: Using `size` aesthetic for lines was deprecated in ggplot2 3.4.0.
#> ℹ Please use `linewidth` instead.
#> This warning is displayed once every 8 hours.
#> Call `lifecycle::last_lifecycle_warnings()` to see where this warning was
#> generated.
#> Warning: The dot-dot notation (`..count..`) was deprecated in ggplot2 3.4.0.
#> ℹ Please use `after_stat(count)` instead.
#> This warning is displayed once every 8 hours.
#> Call `lifecycle::last_lifecycle_warnings()` to see where this warning was
#> generated.
```

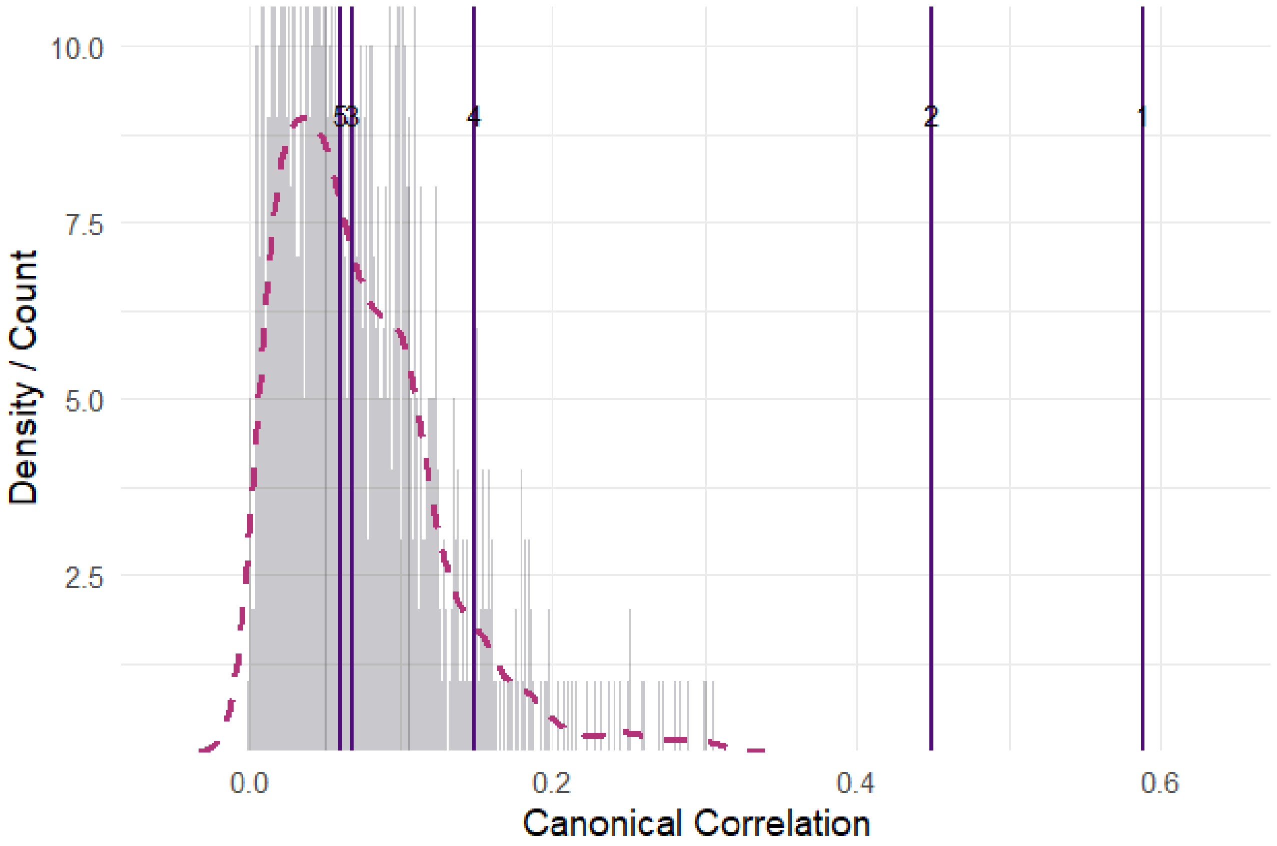


```
#> Empirical p-values:
#> 0
#> 0
#> 0.45
#> 0.083
#> 0.498
#> NULL
```

Figure 12

```
alpha = (sapply(1:K, function(k) res_k[[k]]$alpha))
data_list = list(XX2, YY2)
cpev_toscca = cumsum(sapply(1:K, function(k) cpev.toscca(data_list[[1]], alpha[,k])
if(K>1) {
    auto_cor = stats::cor(alpha)[2:(K+1)]
    adj_cpev_toscca = c(cpev_toscca[1],
                        sapply(2:K, function(k) cpev_toscca[k]*prod(1-abs(auto_cor[
    cat("adjusted cpev is: ", adj_cpev_toscca)

    df <- data.frame(
        K_index = 0:K,
        cpev_toscca = c(0,cpev_toscca),
        adj_cpev_toscca = c(0,adj_cpev_toscca)
```

```
    )
    custom_colors <- c("cpev" = "#5E177F",  # Bright Yellow
                       "adj cpev" = "#F0703C")  # Magenta-Purple
    linetypes <- c("cpev" = "solid",  # Bright Yellow
                   "adj cpev" = "dashed")
    p = ggplot2::ggplot(df, ggplot2::aes(x = K_index)) +
        ggplot2::geom_line(ggplot2::aes(y = cpev_toscca, color = "cpev", linetype =
        ggplot2::geom_line(ggplot2::aes(y = adj_cpev_toscca, color = "adj cpev", li
        ggplot2::scale_color_manual(name = "Legend", values = custom_colors) +  # M
        ggplot2::scale_linetype_manual(name = "Legend", values =linetypes) +  # Set
        ggplot2::scale_x_continuous(breaks = seq(1, K, by = 1)) +  # Force integer
        ggplot2::theme_minimal() +
        ggplot2::labs(
            x = "K",
            y = "%"
        )
    p = p+   ggplot2::ggtitle("cpev toscca") + ggplot2::theme(plot.title = ggplot2:


    print(p)
  }
#> adjusted cpev is:  0.0004697775 0.000546491 0.0005582719 0.0005748263 0.0005238544
```

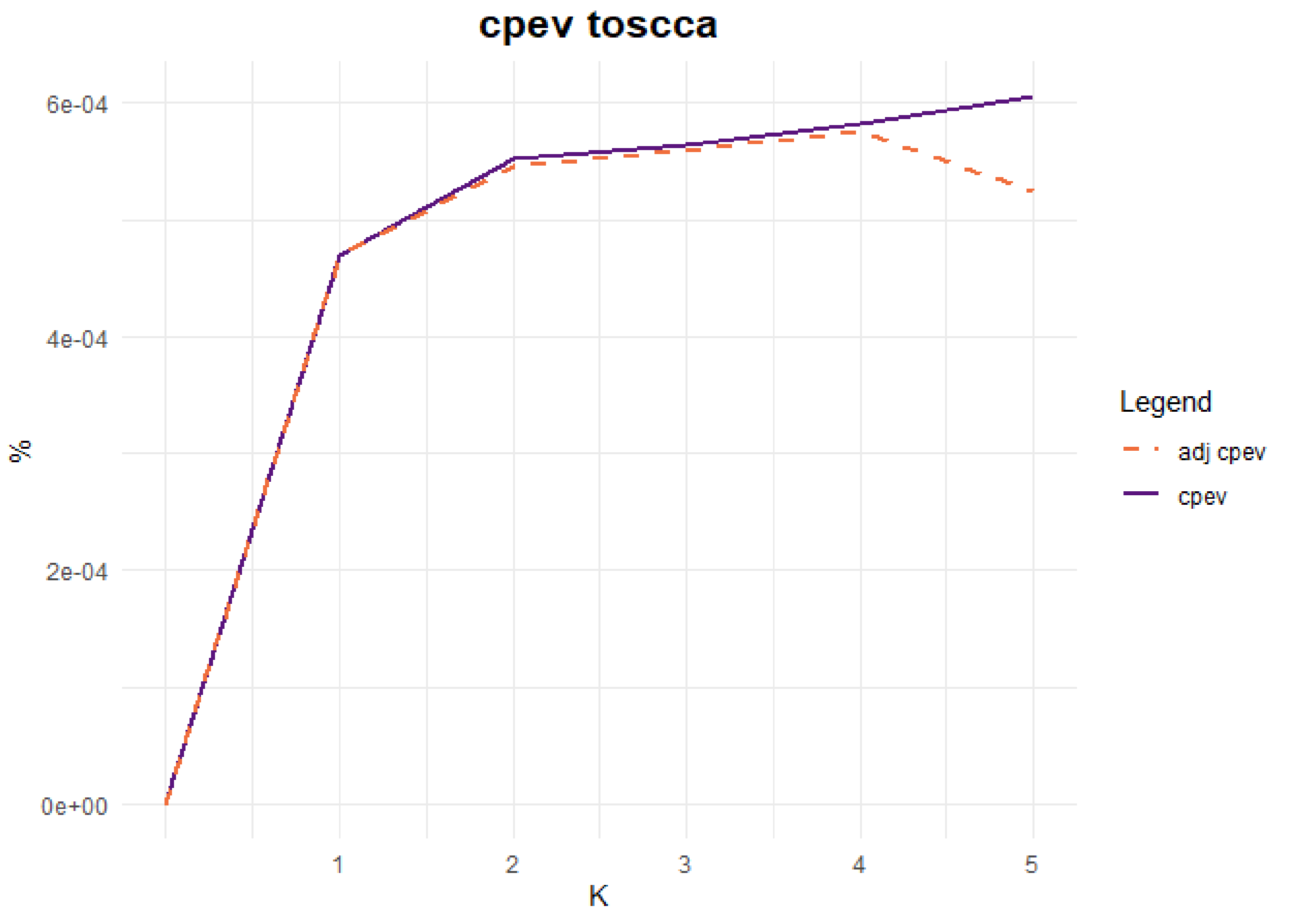

Real data

```
#>
#> Attaching package: 'igraph'
#> The following objects are masked from 'package:stats':
#>
#>     decompose, spectrum
#> The following object is masked from 'package:base':
#>
#>     union
#> ── Attaching core tidyverse packages ──────────────────────── tidyverse 2.0.0 ──
#> ✓ dplyr     1.1.4     ✓ readr     2.1.5
#> ✓ forcats   1.0.0     ✓ stringr   1.5.2
#> ✓ lubridate 1.9.4     ✓ tibble    3.3.0
#> ✓ purrr     1.1.0     ✓ tidyr     1.3.1
#> ── Conflicts ────────────────────────────────────────── tidyverse_conflicts() ──
#> ✕ lubridate::%--%()      masks igraph::%--%()
#> ✕ purrr::accumulate()    masks foreach::accumulate()
#> ✕ dplyr::as_data_frame() masks tibble::as_data_frame(), igraph::as_data_frame()
#> ✕ dplyr::combine()       masks gridExtra::combine()
#> ✕ purrr::compose()       masks igraph::compose()
#> ✕ tidyr::crossing()      masks igraph::crossing()
#> ✕ dplyr::filter()        masks stats::filter()
#> ✕ dplyr::group_rows()    masks kableExtra::group_rows()
#> ✕ dplyr::lag()           masks stats::lag()
#> ✕ purrr::simplify()      masks igraph::simplify()
#> ✕ purrr::when()          masks foreach::when()
#> ℹ Use the conflicted package (<http://conflicted.r-lib.org/>) to force all conflict
#>
#> Attaching package: 'scales'
#>
#>
#> The following object is masked from 'package:purrr':
#>
#>     discard
#>
#>
#> The following object is masked from 'package:readr':
#>
#>     col_factor
#>
#>
#> The following object is masked from 'package:viridis':
#>
#>     viridis_pal
#>
#>
```

```
#>
#> Attaching package: 'plotly'
#>
#>
#> The following object is masked from 'package:igraph':
#>
#>     groups
#>
#>
#> The following object is masked from 'package:ggplot2':
#>
#>     last_plot
#>
#>
#> The following object is masked from 'package:stats':
#>
#>     filter
#>
#>
#> The following object is masked from 'package:graphics':
#>
#>     layout
```

In this section I analyse the Human Microbiome Project data. I will focus on creating highly correlated paths of the gut microbiome and gene expressions.

The gut microbiome is organised in a Taxonomic Tree. This tree groups the microbiota into levels: genus, family, order, class and phylum. In the graph below I have plotted this tree for the available data. Phylums Bacteroides and Firmicutes are the most abundant and diverse.

Figure 5

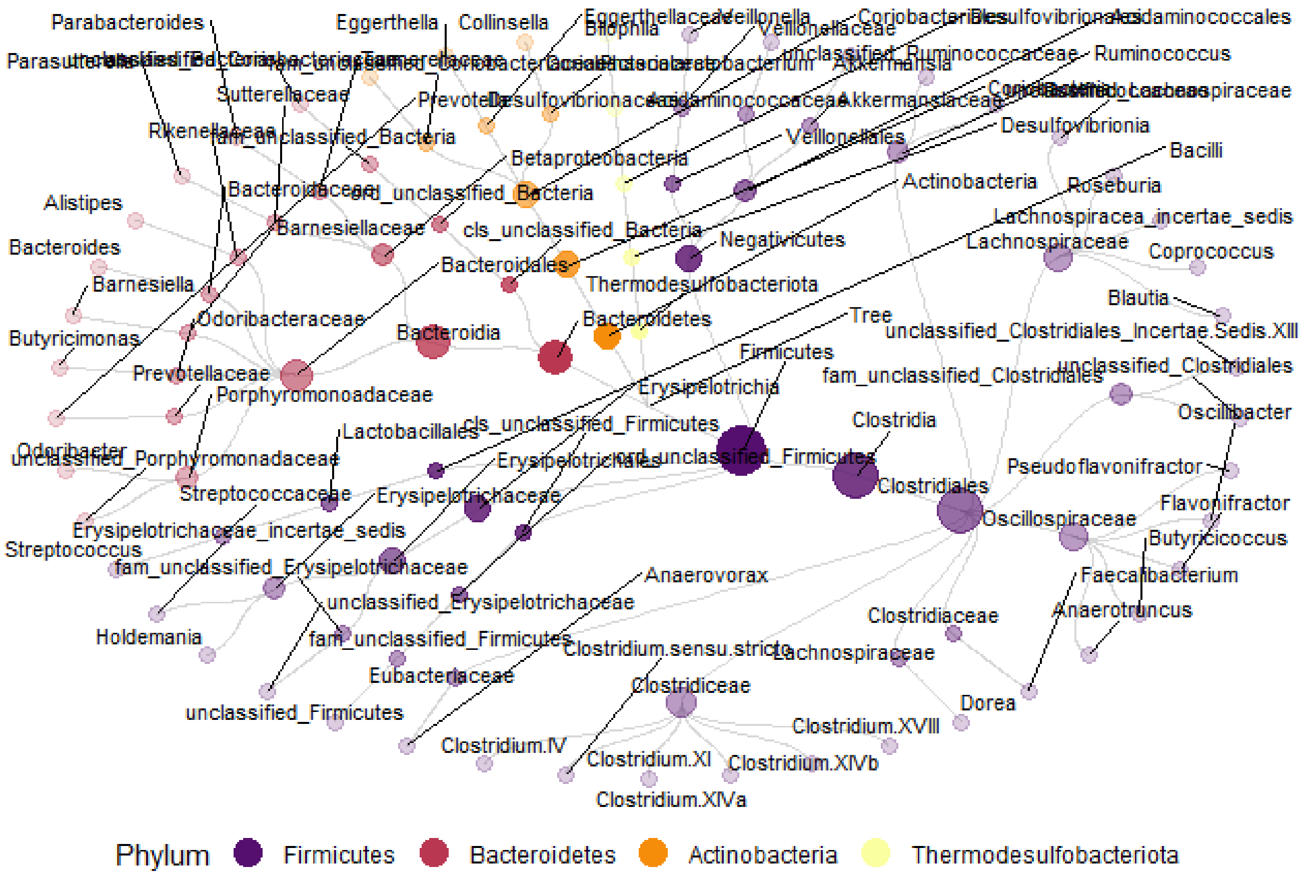

Figure 6

1. Prepare data

We standardise to focus on general changes and not individual stable idiosyncracies.

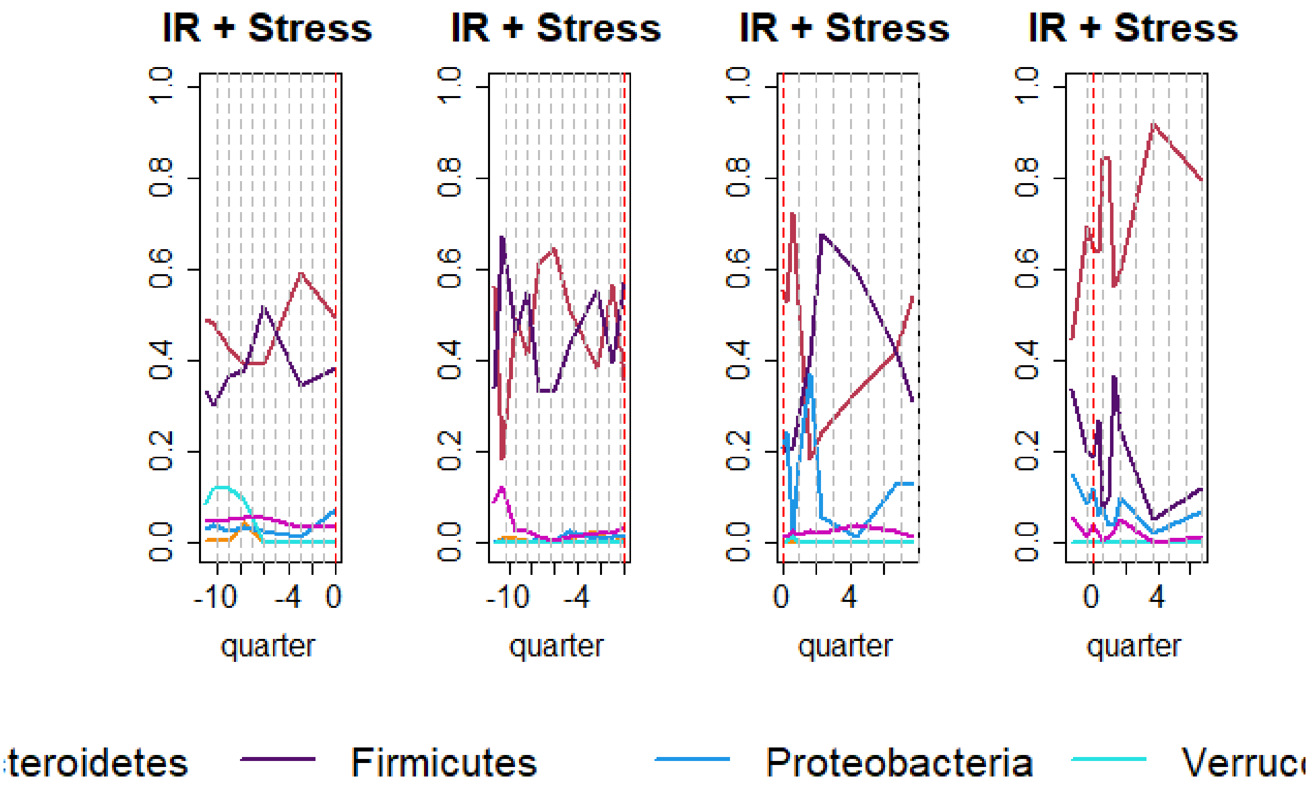

IS group

```
X0 = X0_all[X0_all$id %in% info_hmp[info_hmp$Group == "insulin-sensitive", ]$SubjectI
colnames(X0)[1:2] <-c("id", "time")
Y0 = Y0_all[Y0_all$id %in% info_hmp[info_hmp$Group == "insulin-sensitive", ]$SubjectI
colnames(Y0)[1:2] <-c("id", "time")
# X0 = X0[!is.na(X0$time), ]            # keep selected quarters
# Y0 = Y0[!is.na(Y0$time), ]            # keep selected quarters


# scale and divide data
X0 = scale_rm(X0)
for (level in level_str) {
  Y.temp = data.frame(id = Y0$id, time = Y0$time, Y0[,grep(level, names(Y0))])
  assign(paste0("Y_", substr(level, 1, 3)), scale_rm(Y.temp)); rm(Y.temp)
}


# tosccam -----------------------------------------------------------------------
# set nonzero
mod_stress_control = list()
for (level in level_str) {
  X.temp = X0
```

```
    Y.temp = get(paste0("Y_", substr(level, 1, 3)))

    nz_a_gen = unique(round(seq(from = 5, to = 1000, length.out = 5)))
    nz_b_gen = unique(round(seq(from = 1, to = ncol(Y.temp)-3, length.out = min(8, ncol

    for (k in 1:K) {

      if(k > 1) {
        # residualise for subsequent components
        X.temp = data.frame(X.temp[,c(1,2)],toscca::residualisation(as.matrix(X.temp[,-
        Y.temp = data.frame(Y.temp[,c(1,2)],toscca::residualisation(as.matrix(Y.temp[,-


        # nz_a_gen = as.numeric(table(mod_stress_control[[level]][[k-1]]$alpha[,1] != 0
        # nz_b_gen = as.numeric(table(mod_stress_control[[level]][[k-1]]$beta[,1] != 0)
      }

      mod_stress_control[[level]][[k]] <- tosccamm(X.temp, Y.temp, folds = 2,
                                          nonzero_a = nz_a_gen, nonzero_b = nz_b_gen,
                                          model = "lme", lmeformula = lmeformule, weight_
      if(k == 2 & level == "phylum_") break

    }


    rm(X.temp); rm(Y.temp)
  }
#>  Common convergence error: 0 & Iterations: 4
#>  Common convergence error: 0 & Iterations: 4
#>
#> k-fold cv max. cancor
#>             0.5320024
#>
#>  ........................................
#>  # nonzero A: 254
#>  # nonzero B: 44
#>  ........................................
#>  Common convergence error: 0 & Iterations: 20
#>  Common convergence error: 0.00073 & Iterations: 21
#>
#> k-fold cv max. cancor
#>             0.9308217
#>
#>  ........................................
#>  # nonzero A: 1000
#>  # nonzero B: 7
#>  ........................................
#>  Common convergence error: 0.00265 & Iterations: 21
#>  Common convergence error: 0.00083 & Iterations: 21
```

```
#>
#> k-fold cv max. cancor
#>             0.8498891
#>
#>  ........................................
#>  # nonzero A: 5
#>  # nonzero B: 13
#>  ........................................
#>  Common convergence error: 0 & Iterations: 4
#>  Common convergence error: 0 & Iterations: 4
#>
#> k-fold cv max. cancor
#>             0.6536534
#>
#>  ........................................
#>  # nonzero A: 254
#>  # nonzero B: 17
#>  ........................................
#>  Common convergence error: 0 & Iterations: 21
#>  Common convergence error: 3e-05 & Iterations: 21
#>
#> k-fold cv max. cancor
#>             0.8915907
#>
#>  ........................................
#>  # nonzero A: 1000
#>  # nonzero B: 1
#>  ........................................
#>  Common convergence error: 0.01931 & Iterations: 21
#>  Common convergence error: 0.02036 & Iterations: 21
#>
#> k-fold cv max. cancor
#>             0.9828492
#>
#>  ........................................
#>  # nonzero A: 5
#>  # nonzero B: 17
#>  ........................................
#>  Common convergence error: 0 & Iterations: 4
#>  Common convergence error: 0 & Iterations: 4
#>
#> k-fold cv max. cancor
#>             0.6284839
#>
#>  ........................................
#>  # nonzero A: 254
#>  # nonzero B: 11
#>  ........................................
#>  Common convergence error: 7e-05 & Iterations: 21
```

```
#>  Common convergence error: 0 & Iterations: 21
#>
#> k-fold cv max. cancor
#>            0.9119328
#>
#>  .......................................
#>  # nonzero A: 254
#>  # nonzero B: 1
#>  .......................................
#>  Common convergence error: 1e-05 & Iterations: 21
#>  Common convergence error: 0 & Iterations: 21
#>
#> k-fold cv max. cancor
#>            0.9858612
#>
#>  .......................................
#>  # nonzero A: 254
#>  # nonzero B: 1
#>  .......................................
#>  Common convergence error: 0 & Iterations: 4
#>  Common convergence error: 0 & Iterations: 4
#>
#> k-fold cv max. cancor
#>            0.6296666
#>
#>  .......................................
#>  # nonzero A: 254
#>  # nonzero B: 11
#>  .......................................
#>  Common convergence error: 0.00023 & Iterations: 21
#>  Common convergence error: 0 & Iterations: 19
#>
#> k-fold cv max. cancor
#>            0.9152387
#>
#>  .......................................
#>  # nonzero A: 254
#>  # nonzero B: 1
#>  .......................................
#>  Common convergence error: 1e-05 & Iterations: 21
#>  Common convergence error: 0 & Iterations: 17
#>
#> k-fold cv max. cancor
#>            0.9876397
#>
#>  .......................................
#>  # nonzero A: 254
#>  # nonzero B: 2
#>  .......................................
```

```
#>  Common convergence error: 0 & Iterations: 4
#>  Common convergence error: 0 & Iterations: 4
#>
#> k-fold cv max. cancor
#>             0.5040931
#>
#>  .......................................
#>  # nonzero A: 5
#>  # nonzero B: 2
#>  .......................................
#>  Common convergence error: 0 & Iterations: 18
#>  Common convergence error: 0.00092 & Iterations: 21
#>
#> k-fold cv max. cancor
#>             0.9431128
#>
#>  .......................................
#>  # nonzero A: 254
#>  # nonzero B: 1
#>  .......................................
```

```
for (level in level_str) {

  if(level == "phylum_") K = 2

  # canonical vectors
  cv.temp = matrix(NA, nrow = ncol(X0)-2, ncol = K)
  cv.temp = sapply(1:K, function(k) cbind(mod_stress_control[[level]][[k]]$alpha[,1])
  rownames(cv.temp) <- colnames(X0[,-c(1,2)])
  assign(paste0("alpha_control_", substr(level, 1, 3)), cv.temp)

  cv.temp = matrix(NA, nrow = ncol(get(paste0("Y_", substr(level, 1, 3))))-2, ncol =
  cv.temp = sapply(1:K, function(k) cbind(mod_stress_control[[level]][[k]]$beta[,1]))
  rownames(cv.temp) <- colnames(get(paste0("Y_", substr(level, 1, 3)))[,-c(1,2)])
  assign(paste0("beta_control_", substr(level, 1, 3)), cv.temp); rm(cv.temp)

  # latent variables
  lv.temp = sapply(1:K, function(k) as.matrix(X0[,-c(1,2)]) %*% get(paste0("alpha_con
  df.temp = data.frame(id = X0$id, time = X0$time, lv.temp)
  assign(paste0("lv_a_control_", substr(level, 1, 3)), df.temp); rm(lv.temp); rm(df.t

  lv.temp = sapply(1:K, function(k) as.matrix(get(paste0("Y_", substr(level,1,3)))[,-
  df.temp =  data.frame(id = (get(paste0("Y_", substr(level, 1, 3))))$id, time = (get
  assign(paste0("lv_b_control_", substr(level, 1, 3)), df.temp); rm(lv.temp); rm(df.t

}
```

IR group

```
K=3
X0 = X0_all[X0_all$id %in% info_hmp[info_hmp$Group %in% "insulin-resistant", ]$Subjec
colnames(X0)[1:2] <-c("id", "time")
Y0 = Y0_all[Y0_all$id %in% info_hmp[info_hmp$Group %in% "insulin-resistant", ]$Subjec
colnames(Y0)[1:2] <-c("id", "time")
# X0 = X0[!is.na(X0$time), ]             # keep selected quarters
# Y0 = Y0[!is.na(Y0$time), ]             # keep selected quarters


# scale and divide data
X0 = scale_rm(X0)
for (level in level_str) {
  Y.temp = data.frame(id = Y0$id, time = Y0$time, Y0[,grep(level, names(Y0))])
  assign(paste0("Y_", substr(level, 1, 3)), scale_rm(Y.temp)); rm(Y.temp)
}

mod_stress_predDB = list()
for (level in level_str) {
  X.temp = X0
  Y.temp = get(paste0("Y_", substr(level, 1, 3)))

  nz_a_gen = unique(round(seq(from = 5, to = 1000, length.out = 9)))
  nz_b_gen = unique(round(seq(from = 1, to = ncol(Y.temp)-3, length.out = min(9, ncol

  for (k in 1:K) {

    if(k > 1) {
      # residualise for subsequent components
      X.temp = data.frame(X.temp[,c(1,2)],toscca::residualisation(as.matrix(X.temp[,-
      Y.temp = data.frame(Y.temp[,c(1,2)],toscca::residualisation(as.matrix(Y.temp[,-


      # nz_a_gen = as.numeric(table(mod_stress_predDB[[level]][[k-1]]$alpha[,1] != 0)
      # nz_b_gen = as.numeric(table(mod_stress_predDB[[level]][[k-1]]$beta[,1] != 0)[
    }

    mod_stress_predDB[[level]][[k]] <- tosccamm(X.temp, Y.temp, folds = 2,
                                       nonzero_a = nz_a_gen, nonzero_b = nz_b_gen,
                                       model = "lme", lmeformula = lmeformule, weight_
    if(k == 2 & level == "phylum_") break

  }


  rm(X.temp); rm(Y.temp)
}
#>  Common convergence error: 0 & Iterations: 4
#>  Common convergence error: 0 & Iterations: 4
```

```
#>
#> k-fold cv max. cancor
#>             0.8317791
#>
#>  ........................................
#>  # nonzero A: 129
#>  # nonzero B: 17
#>  ........................................
#>  Common convergence error: 0.00046 & Iterations: 21
#>  Common convergence error: 3e-05 & Iterations: 21
#>
#> k-fold cv max. cancor
#>             0.7768828
#>
#>  ........................................
#>  # nonzero A: 1000
#>  # nonzero B: 1
#>  ........................................
#>  Common convergence error: 7e-04 & Iterations: 21
#>  Common convergence error: 0.0157 & Iterations: 21
#>
#> k-fold cv max. cancor
#>              0.741953
#>
#>  ........................................
#>  # nonzero A: 751
#>  # nonzero B: 12
#>  ........................................
#>  Common convergence error: 0 & Iterations: 4
#>  Common convergence error: 0 & Iterations: 4
#>
#> k-fold cv max. cancor
#>             0.8489665
#>
#>  ........................................
#>  # nonzero A: 254
#>  # nonzero B: 6
#>  ........................................
#>  Common convergence error: 2e-05 & Iterations: 21
#>  Common convergence error: 0.00053 & Iterations: 21
#>
#> k-fold cv max. cancor
#>             0.8124422
#>
#>  ........................................
#>  # nonzero A: 5
#>  # nonzero B: 3
#>  ........................................
#>  Common convergence error: 2e-05 & Iterations: 21
```

```
#>  Common convergence error: 2e-05 & Iterations: 21
#>
#> k-fold cv max. cancor
#>            0.7303294
#>
#>  ........................................
#>  # nonzero A: 5
#>  # nonzero B: 1
#>  ........................................
#>  Common convergence error: 0 & Iterations: 4
#>  Common convergence error: 0 & Iterations: 4
#>
#> k-fold cv max. cancor
#>            0.7421304
#>
#>  ........................................
#>  # nonzero A: 254
#>  # nonzero B: 6
#>  ........................................
#>  Common convergence error: 0.01739 & Iterations: 21
#>  Common convergence error: 0.00519 & Iterations: 21
#>
#> k-fold cv max. cancor
#>            0.7936958
#>
#>  ........................................
#>  # nonzero A: 5
#>  # nonzero B: 10
#>  ........................................
#>  Common convergence error: 0.00036 & Iterations: 21
#>  Common convergence error: 0.01001 & Iterations: 21
#>
#> k-fold cv max. cancor
#>            0.9237112
#>
#>  ........................................
#>  # nonzero A: 129
#>  # nonzero B: 7
#>  ........................................
#>  Common convergence error: 0 & Iterations: 4
#>  Common convergence error: 0 & Iterations: 4
#>
#> k-fold cv max. cancor
#>            0.7427633
#>
#>  ........................................
#>  # nonzero A: 254
#>  # nonzero B: 6
#>  ........................................
```

```
#>  Common convergence error: 0.01588 & Iterations: 21
#>  Common convergence error: 0.00464 & Iterations: 21
#>
#> k-fold cv max. cancor
#>             0.7932756
#>
#>  .......................................
#>  # nonzero A: 5
#>  # nonzero B: 10
#>  .......................................
#>  Common convergence error: 0.00036 & Iterations: 21
#>  Common convergence error: 0.00313 & Iterations: 21
#>
#> k-fold cv max. cancor
#>             0.9221328
#>
#>  .......................................
#>  # nonzero A: 129
#>  # nonzero B: 10
#>  .......................................
#>  Common convergence error: 0 & Iterations: 4
#>  Common convergence error: 0 & Iterations: 4
#>
#> k-fold cv max. cancor
#>             0.7104961
#>
#>  .......................................
#>  # nonzero A: 254
#>  # nonzero B: 3
#>  .......................................
#>  Common convergence error: 0.00047 & Iterations: 21
#>  Common convergence error: 0.00668 & Iterations: 21
#>
#> k-fold cv max. cancor
#>             0.6381623
#>
#>  .......................................
#>  # nonzero A: 502
#>  # nonzero B: 1
#>  .......................................
```

```
for (level in level_str) {

  if(level == "phylum_") K = 2

  # canonical vectors
  cv.temp = matrix(NA, nrow = ncol(X0)-2, ncol = K)
  cv.temp = sapply(1:K, function(k) cbind(mod_stress_predDB[[level]][[k]]$alpha[,1]))
  rownames(cv.temp) <- colnames(X0[,-c(1,2)])
```

```
    assign(paste0("alpha_predDB_", substr(level, 1, 3)), cv.temp)

    cv.temp = matrix(NA, nrow = ncol(get(paste0("Y_", substr(level, 1, 3))))-2, ncol =
    cv.temp = sapply(1:K, function(k) cbind(mod_stress_predDB[[level]][[k]]$beta[,1]))
    rownames(cv.temp) <- colnames(get(paste0("Y_", substr(level, 1, 3)))[,-c(1,2)])
    assign(paste0("beta_predDB_", substr(level, 1, 3)), cv.temp); rm(cv.temp)

    # latent variables
    lv.temp = sapply(1:K, function(k) as.matrix(X0[,-c(1,2)]) %*% get(paste0("alpha_pre
    df.temp = data.frame(id = X0$id, time = X0$time, lv.temp)
    assign(paste0("lv_a_predDB_", substr(level, 1, 3)), df.temp); rm(lv.temp); rm(df.te

    lv.temp = sapply(1:K, function(k) as.matrix(get(paste0("Y_", substr(level,1,3)))[,-
    df.temp =  data.frame(id = (get(paste0("Y_", substr(level, 1, 3))))$id, time = (get
    assign(paste0("lv_b_predDB_", substr(level, 1, 3)), df.temp); rm(lv.temp); rm(df.te

  }
```

Plot cpev

figure 8

```
  K=3
  res_k =  mod_stress_predDB[["family_"]]
    beta = (sapply(1:K, function(k) res_k[[k]]$beta))
    data_list = list(Y_fam, X0)
    cpev_toscca = cumsum(sapply(1:K, function(k) cpev.toscca(data_list[[1]], beta[,k]))
    if(K>1) {
        auto_cor = stats::cor(alpha)[2:(K+1)]
        adj_cpev_toscca = c(cpev_toscca[1],
                            sapply(2:K, function(k) cpev_toscca[k]*prod(1-abs(auto_cor[
        cat("adjusted cpev is: ", adj_cpev_toscca)

        df <- data.frame(
            K_index = 0:K,
            cpev_toscca = c(0,cpev_toscca),
            adj_cpev_toscca = c(0,adj_cpev_toscca)
        )
        custom_colors <- c("cpev" = "#5E177F",  # Bright Yellow
                           "adj cpev" = "#F0703C")  # Magenta-Purple
        linetypes <- c("cpev" = "solid",  # Bright Yellow
                       "adj cpev" = "dashed")
        p = ggplot2::ggplot(df, ggplot2::aes(x = K_index)) +
            ggplot2::geom_line(ggplot2::aes(y = cpev_toscca, color = "cpev", linetype =
            ggplot2::geom_line(ggplot2::aes(y = adj_cpev_toscca, color = "adj cpev", li
```

```
        ggplot2::scale_color_manual(name = "Legend", values = custom_colors) +  # M
        ggplot2::scale_linetype_manual(name = "Legend", values =linetypes) +  # Set
        ggplot2::scale_x_continuous(breaks = seq(1, K, by = 1)) +  # Force integer
        ggplot2::theme_minimal() +
        ggplot2::labs(
            x = "K",
            y = "%"
        )
    p = p+   ggplot2::ggtitle("cpev toscca") + ggplot2::theme(plot.title = ggplot2:


    print(p)
}
#> adjusted cpev is:  0.02064692 0.02505975 0.02783344
```

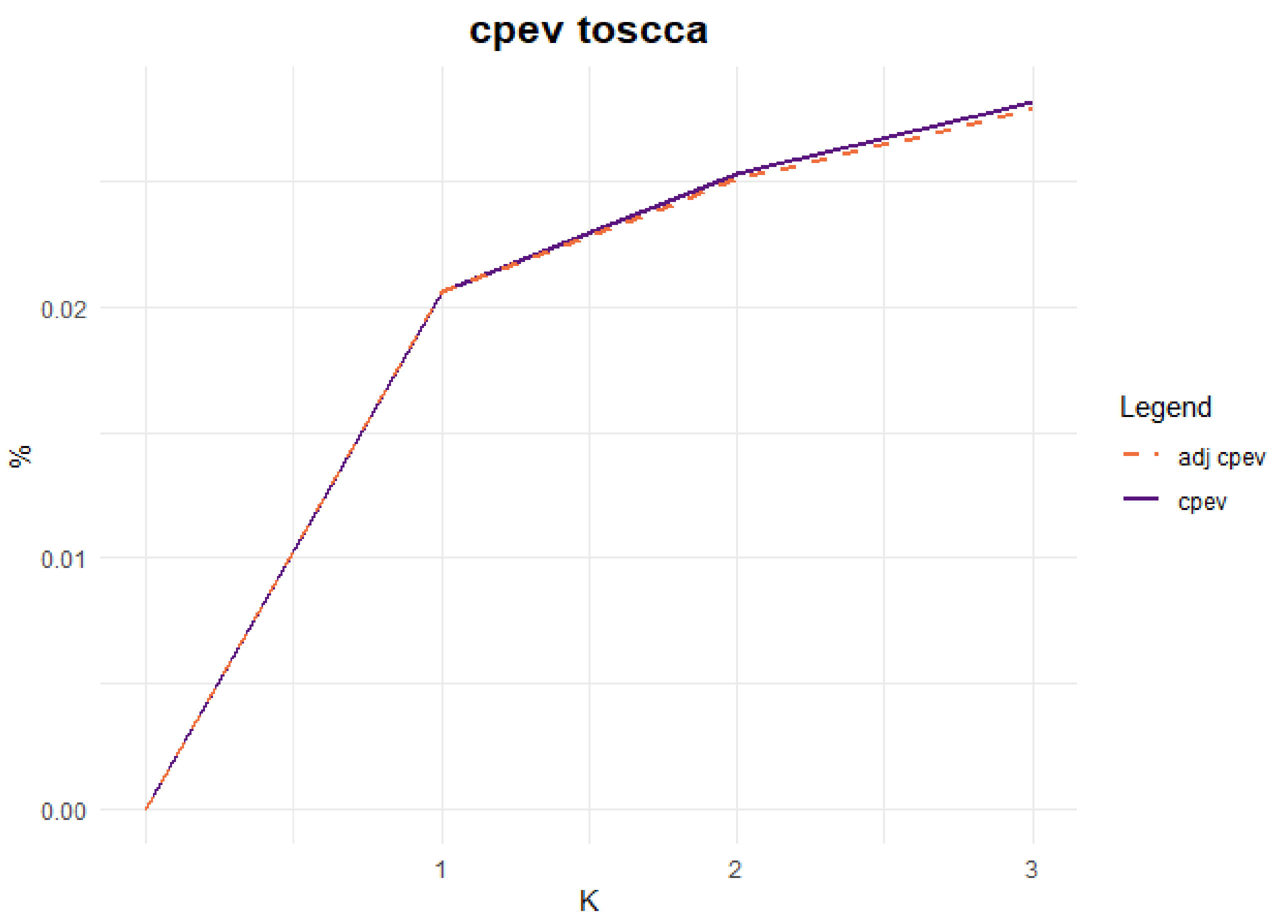

Plot latent variables figure 9 - family level

```
#> `geom_smooth()` using method = 'loess' and formula = 'y ~ x'
#> `geom_smooth()` using method = 'loess' and formula = 'y ~ x'
#> Warning: Removed 1 row containing non-finite outside the scale range
#> (`stat_smooth()`).
#> `geom_smooth()` using method = 'loess' and formula = 'y ~ x'
#> `geom_smooth()` using method = 'loess' and formula = 'y ~ x'
```

```
#> `geom_smooth()` using method = 'loess' and formula = 'y ~ x'
#> `geom_smooth()` using method = 'loess' and formula = 'y ~ x'
#> `geom_smooth()` using method = 'loess' and formula = 'y ~ x'
#> Warning: Removed 28 rows containing non-finite outside the scale range
#> (`stat_smooth()`).
#> `geom_smooth()` using method = 'loess' and formula = 'y ~ x'
```

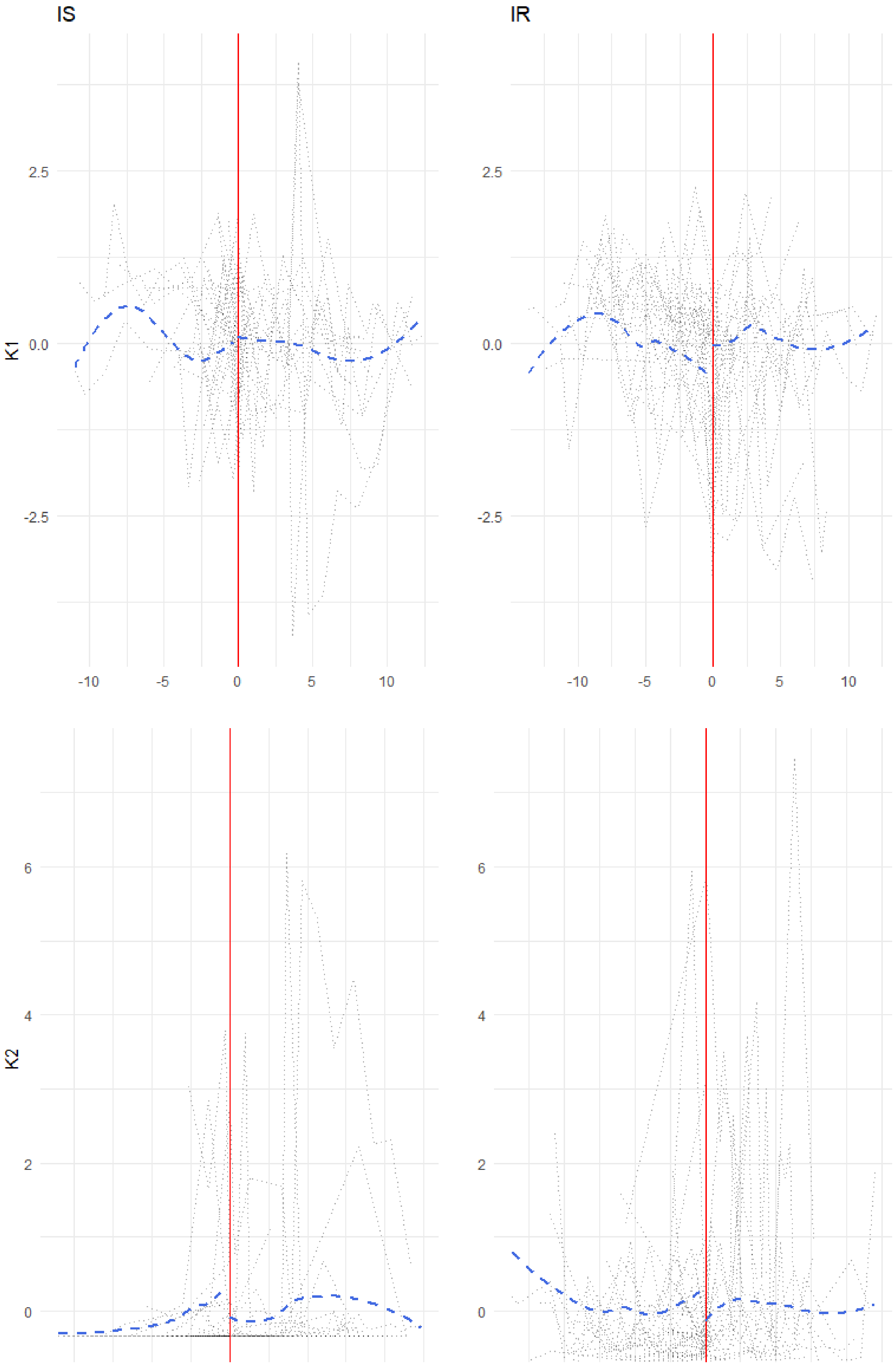
IS
IR
K1
2.5
0.0
-2.5
-10
-5
0
5
10
K2
6
4
2
0

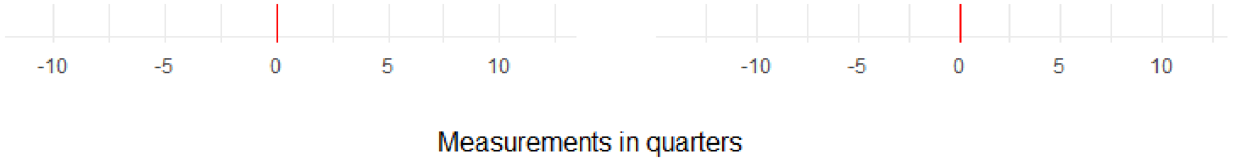

figure 14 - genus level

```
#> `geom_smooth()` using method = 'loess' and formula = 'y ~ x'
#> `geom_smooth()` using method = 'loess' and formula = 'y ~ x'
#> Warning: Removed 1 row containing non-finite outside the scale range
#> (`stat_smooth()`).
#> `geom_smooth()` using method = 'loess' and formula = 'y ~ x'
#> Warning: Removed 2 rows containing non-finite outside the scale range
#> (`stat_smooth()`).
#> `geom_smooth()` using method = 'loess' and formula = 'y ~ x'
#> `geom_smooth()` using method = 'loess' and formula = 'y ~ x'
#> Warning: Removed 1 row containing non-finite outside the scale range
#> (`stat_smooth()`).
#> `geom_smooth()` using method = 'loess' and formula = 'y ~ x'
#> `geom_smooth()` using method = 'loess' and formula = 'y ~ x'
#> Warning: Removed 1 row containing non-finite outside the scale range
#> (`stat_smooth()`).
#> `geom_smooth()` using method = 'loess' and formula = 'y ~ x'
```

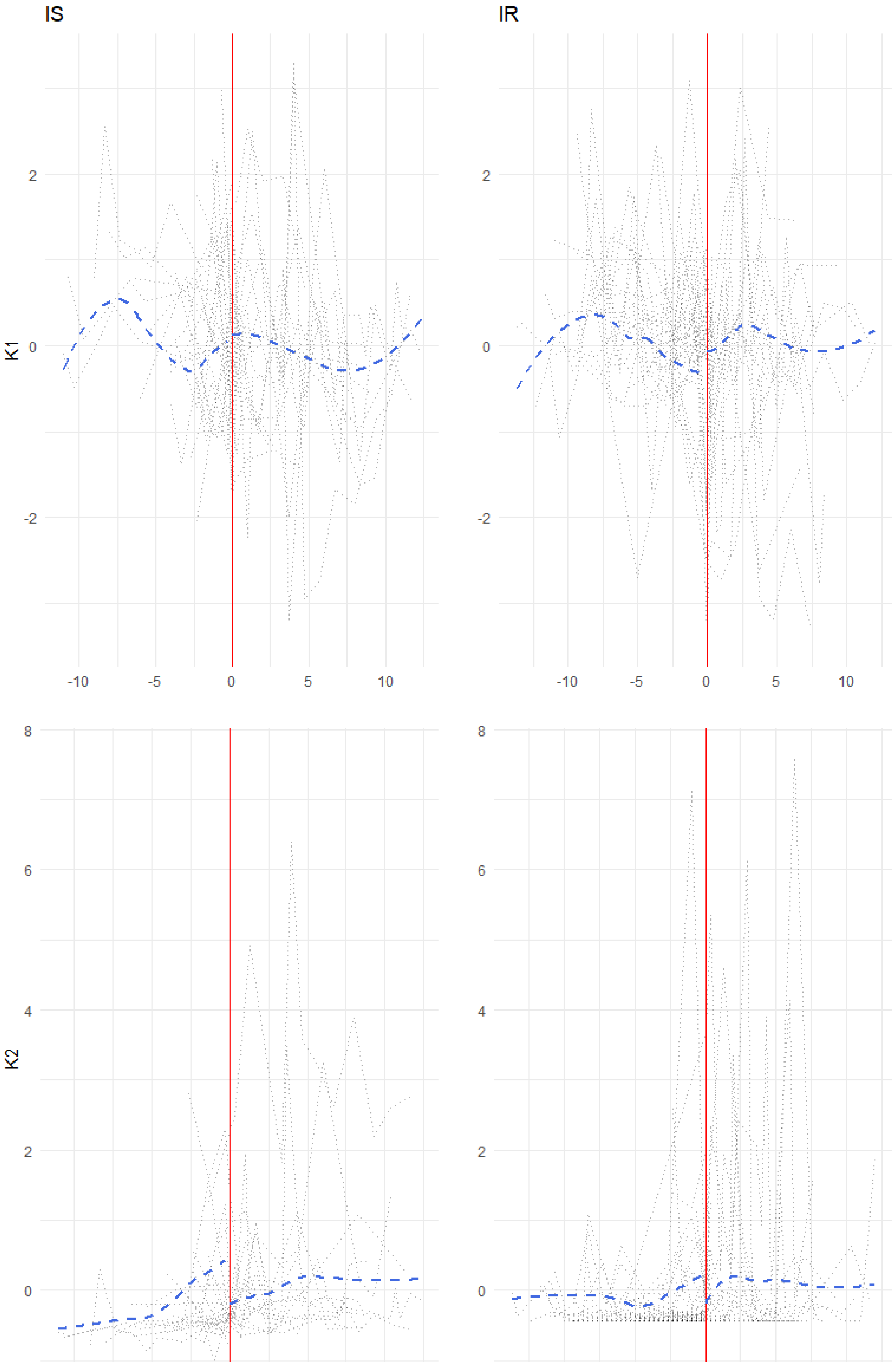
IS
IR
K1
K2
2
0
-2
-10
-5
0
5
10
8
6
4

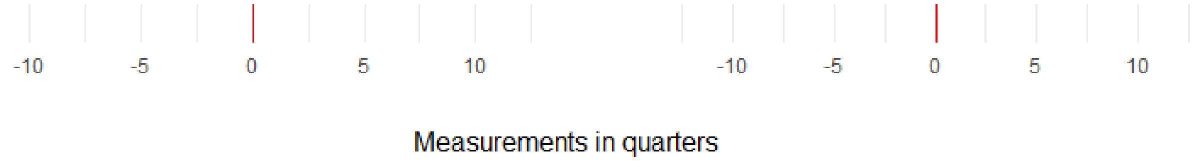

figure 15 - order level

```
#> `geom_smooth()` using method = 'loess' and formula = 'y ~ x'
#> `geom_smooth()` using method = 'loess' and formula = 'y ~ x'
#> Warning: Removed 1 row containing non-finite outside the scale range
#> (`stat_smooth()`).
#> `geom_smooth()` using method = 'loess' and formula = 'y ~ x'
#> `geom_smooth()` using method = 'loess' and formula = 'y ~ x'
#> Warning: Removed 1 row containing non-finite outside the scale range
#> (`stat_smooth()`).
#> `geom_smooth()` using method = 'loess' and formula = 'y ~ x'
#> `geom_smooth()` using method = 'loess' and formula = 'y ~ x'
#> Warning: Removed 1 row containing non-finite outside the scale range
#> (`stat_smooth()`).
#> `geom_smooth()` using method = 'loess' and formula = 'y ~ x'
#> `geom_smooth()` using method = 'loess' and formula = 'y ~ x'
```

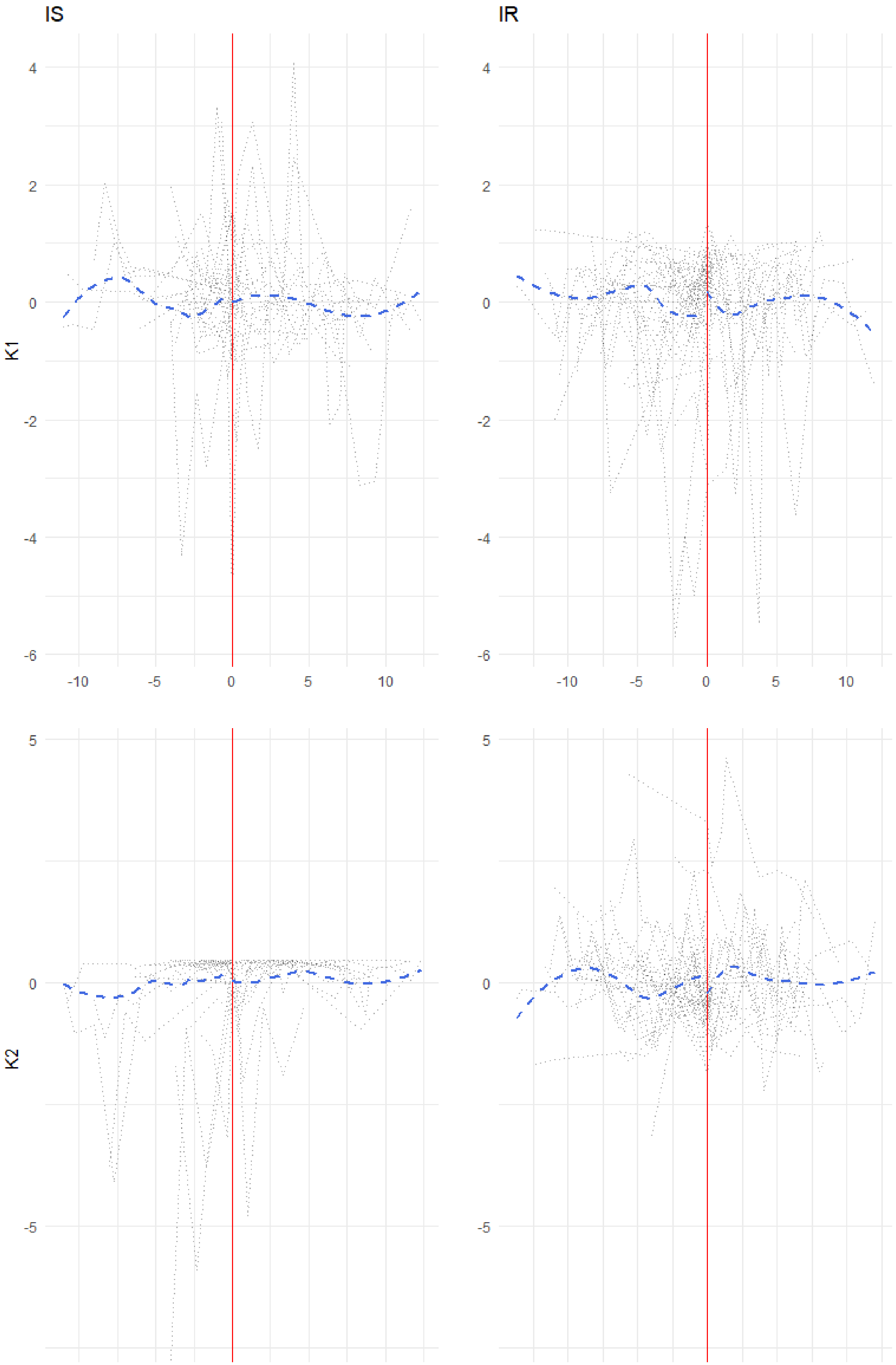
IS
IR
K1
K2
4
2
0
-2
-4
-6
-10
-5
0
5
10
5
0
-5

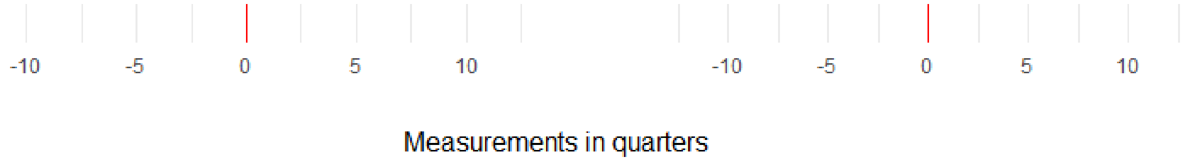

figure 16 - class level

```
#> `geom_smooth()` using method = 'loess' and formula = 'y ~ x'
#> `geom_smooth()` using method = 'loess' and formula = 'y ~ x'
#> Warning: Removed 1 row containing non-finite outside the scale range
#> (`stat_smooth()`).
#> `geom_smooth()` using method = 'loess' and formula = 'y ~ x'
#> `geom_smooth()` using method = 'loess' and formula = 'y ~ x'
#> Warning: Removed 1 row containing non-finite outside the scale range
#> (`stat_smooth()`).
#> `geom_smooth()` using method = 'loess' and formula = 'y ~ x'
#> `geom_smooth()` using method = 'loess' and formula = 'y ~ x'
#> Warning: Removed 1 row containing non-finite outside the scale range
#> (`stat_smooth()`).
#> `geom_smooth()` using method = 'loess' and formula = 'y ~ x'
#> `geom_smooth()` using method = 'loess' and formula = 'y ~ x'
```

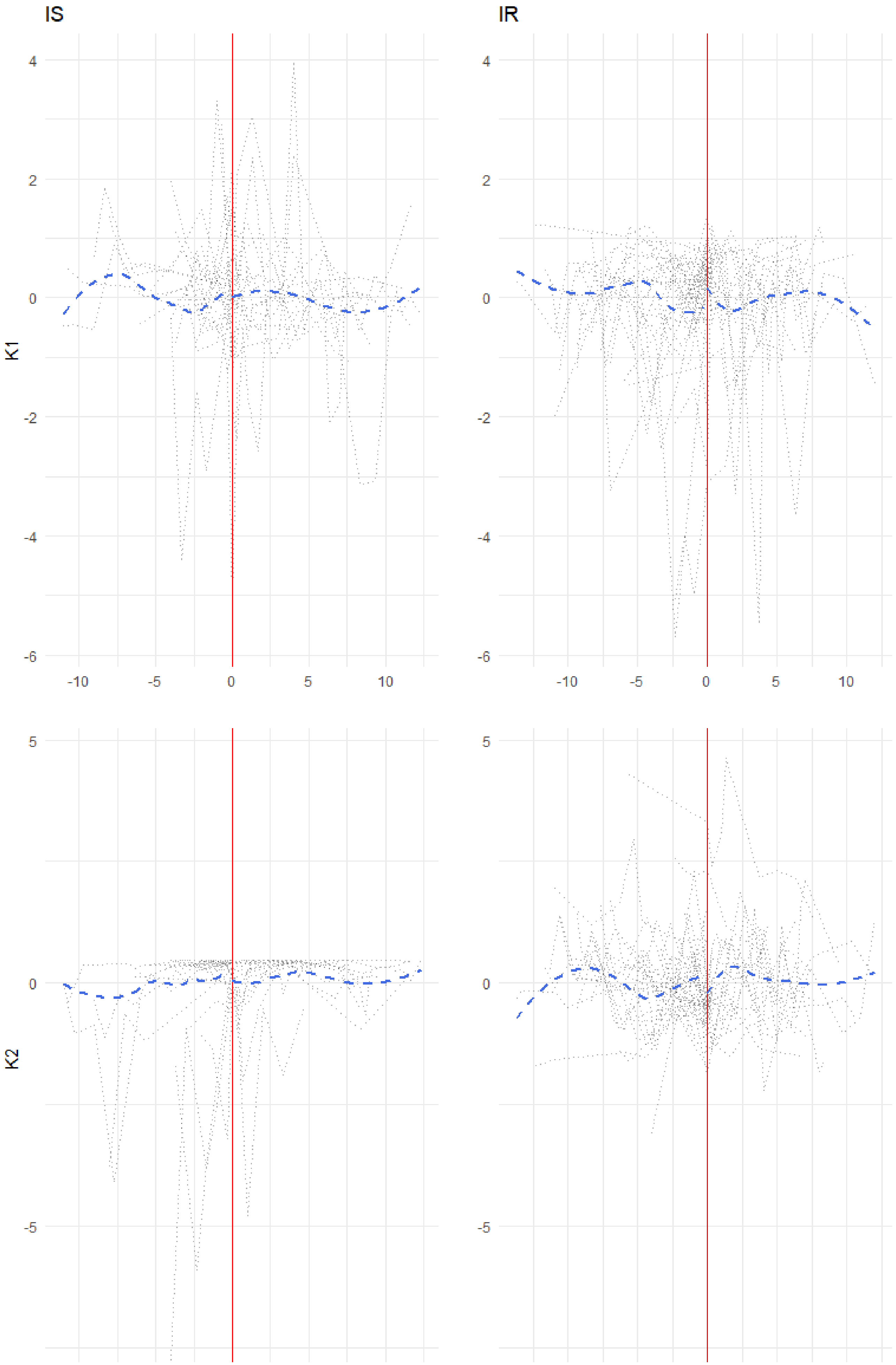
IS
IR
K1
K2
4
2
0
-2
-4
-6
5
0
-5
-10
-5
0
5
10

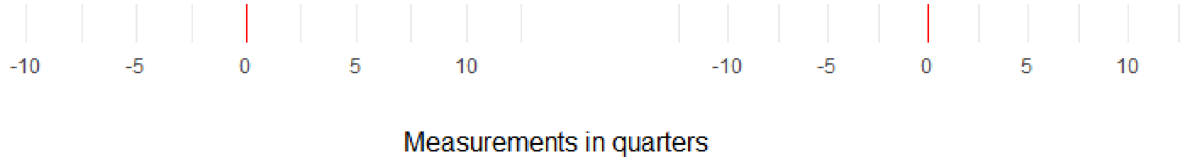

figure 17 - phylum level

```
#> `geom_smooth()` using method = 'loess' and formula = 'y ~ x'
#> `geom_smooth()` using method = 'loess' and formula = 'y ~ x'
#> `geom_smooth()` using method = 'loess' and formula = 'y ~ x'
#> `geom_smooth()` using method = 'loess' and formula = 'y ~ x'
#> Warning: Removed 1 row containing non-finite outside the scale range
#> (`stat_smooth()`).
#> `geom_smooth()` using method = 'loess' and formula = 'y ~ x'
#> `geom_smooth()` using method = 'loess' and formula = 'y ~ x'
#> Warning: Removed 1 row containing non-finite outside the scale range
#> (`stat_smooth()`).
#> `geom_smooth()` using method = 'loess' and formula = 'y ~ x'
#> `geom_smooth()` using method = 'loess' and formula = 'y ~ x'
```

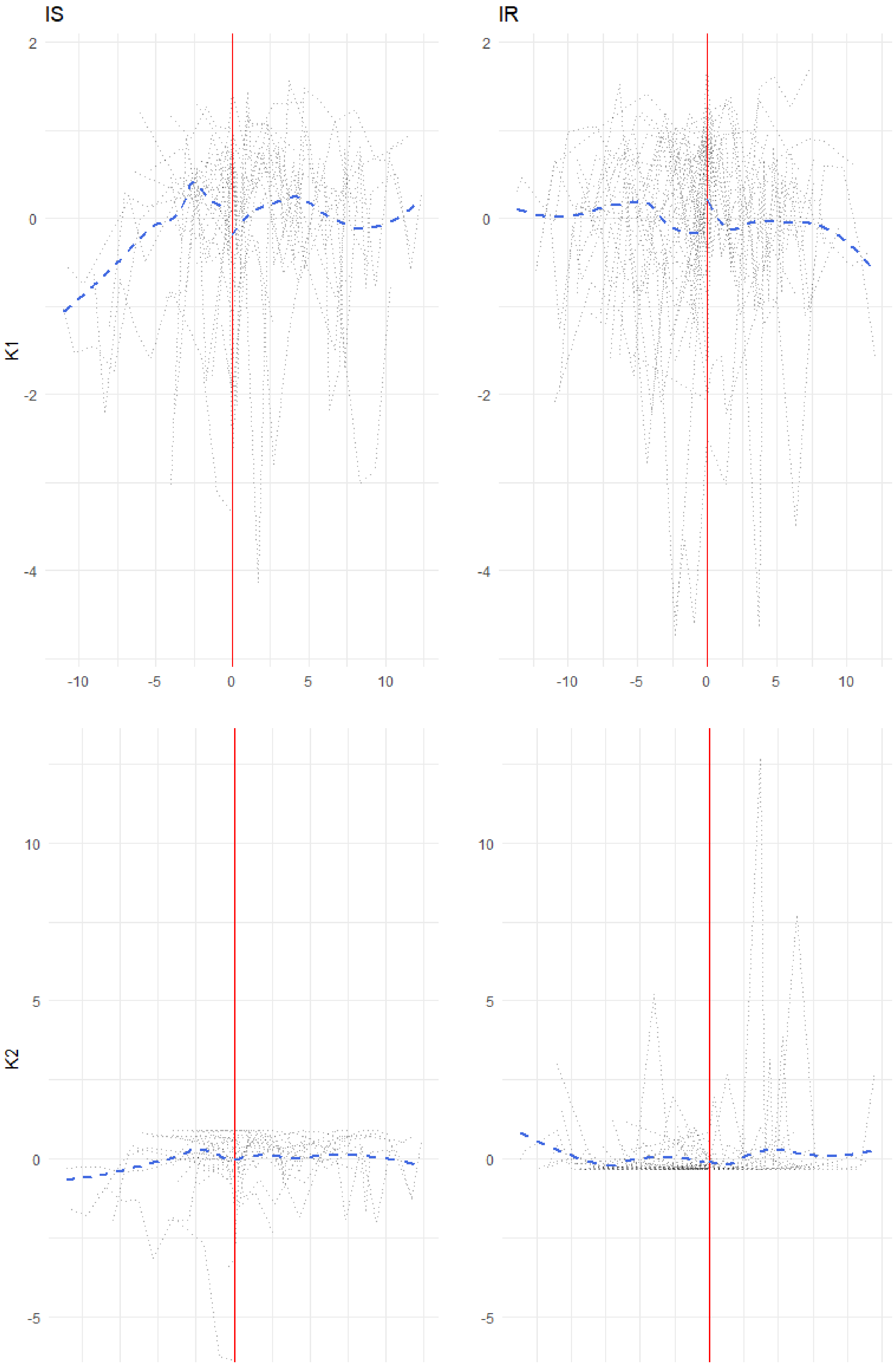
IS
IR
K1
K2
2
0
-2
-4
-10
-5
0
5
10
10
5
0
-5

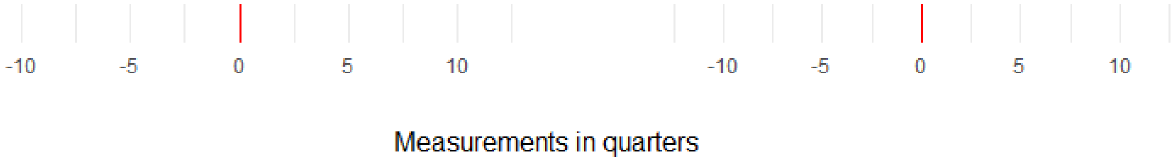
-10
-5
0
5
10
-10
-5
0
5
10
Measurements in quarters
