# Supplementary figures and images for: Sparse Canonical Correlation Analysis for Multiple Measurements With Latent Trajectories
Source: Biom J. 2025 Oct 30;67(6):e70090. doi: 10.1002/bimj.70090 (PMC12573309; doi:10.1002/bimj.70090)

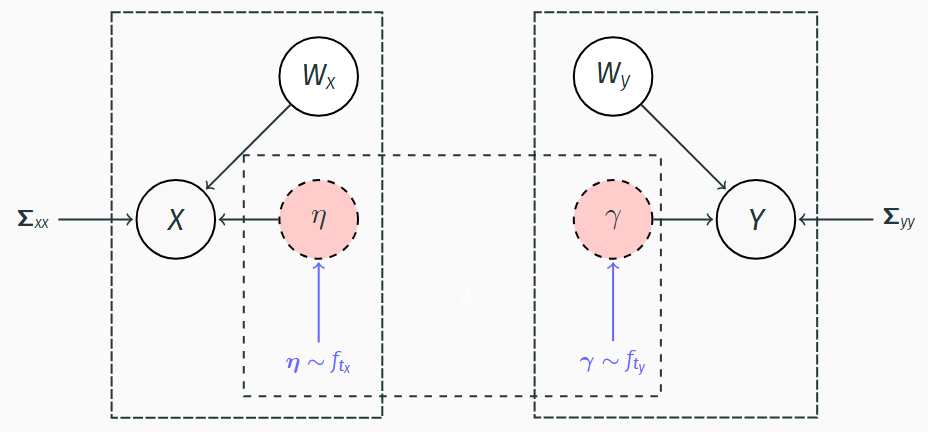

Supplement: Supplementary file 1 — Supporting Information [file BIMJ-67-e70090-s001.zip › tosccammCode_senar/toscca-mm-main/diagram/tosccamm_tikz.png]

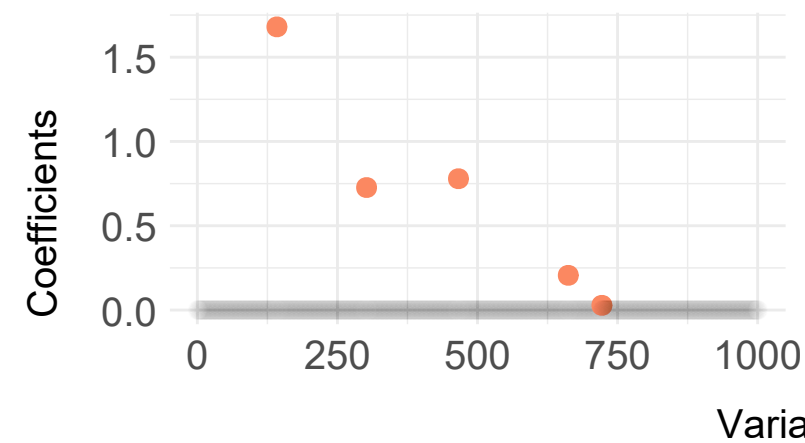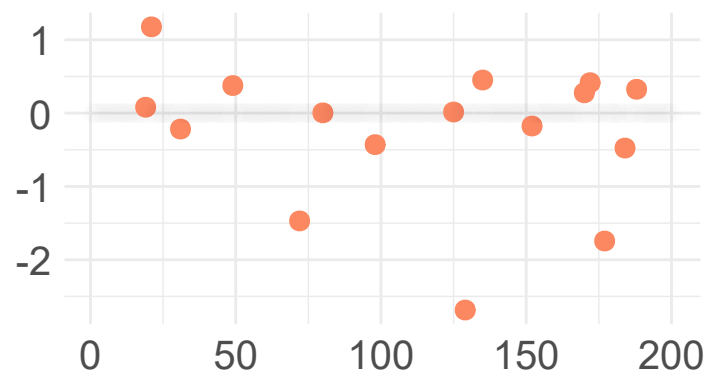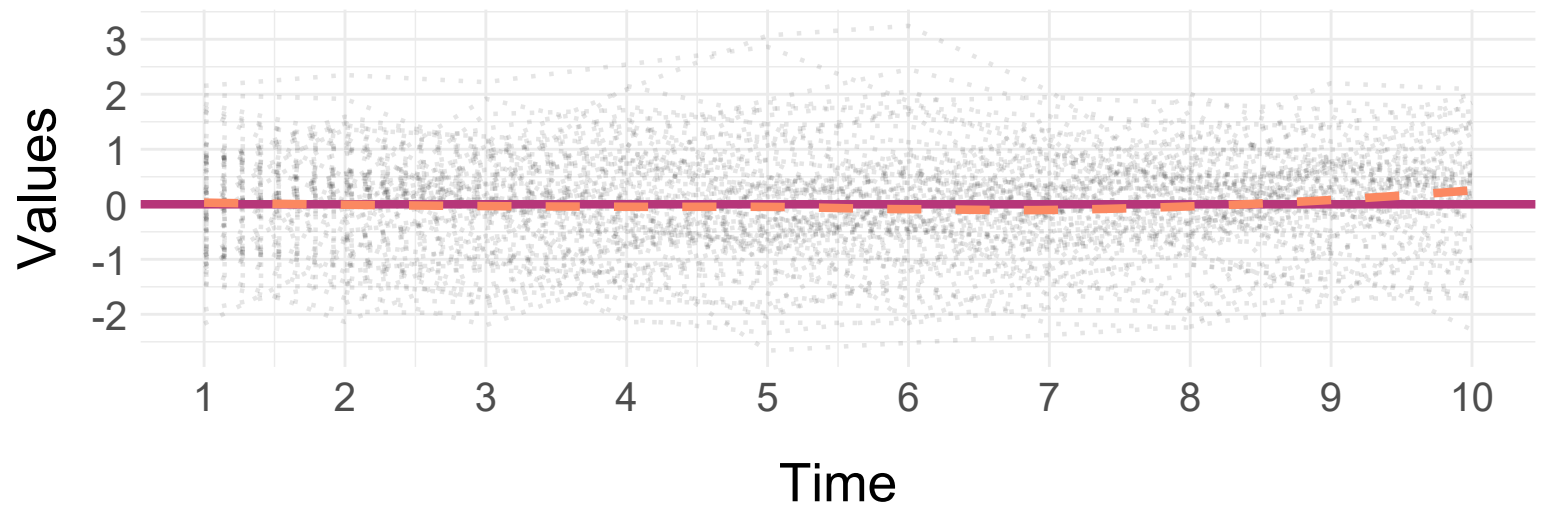

Supplement: Supplementary file 1 — Supporting Information [file BIMJ-67-e70090-s001.zip › tosccammCode_senar/toscca-mm-main/figures/figure_10.pdf]

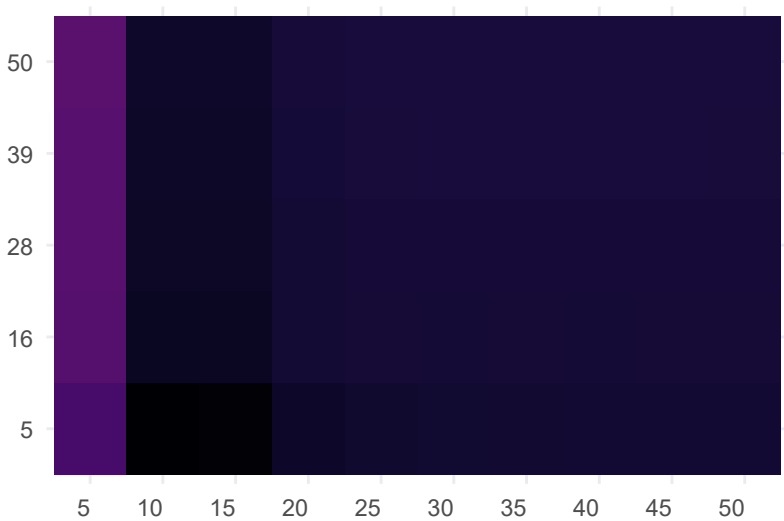

Supplement: Supplementary file 1 — Supporting Information [file BIMJ-67-e70090-s001.zip › tosccammCode_senar/toscca-mm-main/figures/figure_11_a.pdf]

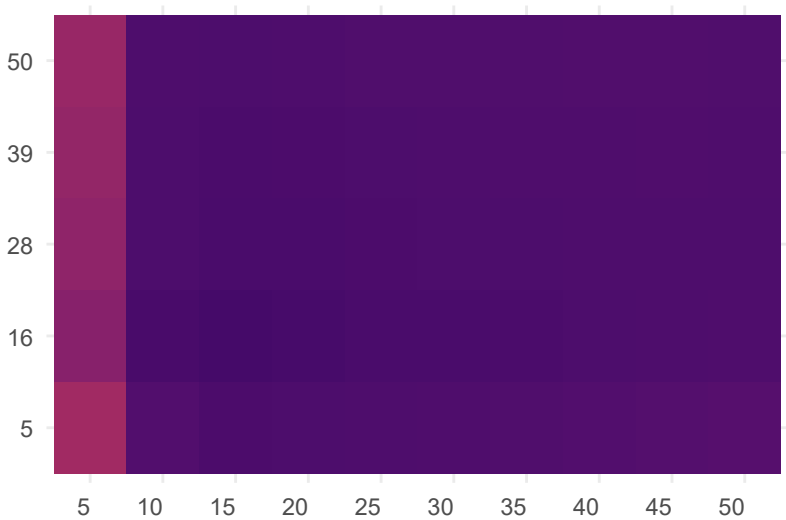

Supplement: Supplementary file 1 — Supporting Information [file BIMJ-67-e70090-s001.zip › tosccammCode_senar/toscca-mm-main/figures/figure_11_b.pdf]

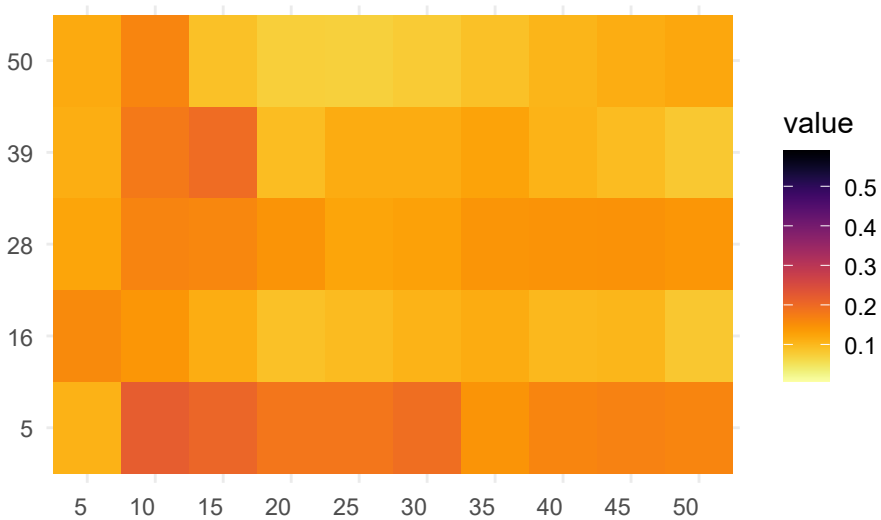

Supplement: Supplementary file 1 — Supporting Information [file BIMJ-67-e70090-s001.zip › tosccammCode_senar/toscca-mm-main/figures/figure_11_c.pdf]

# cpev toscca

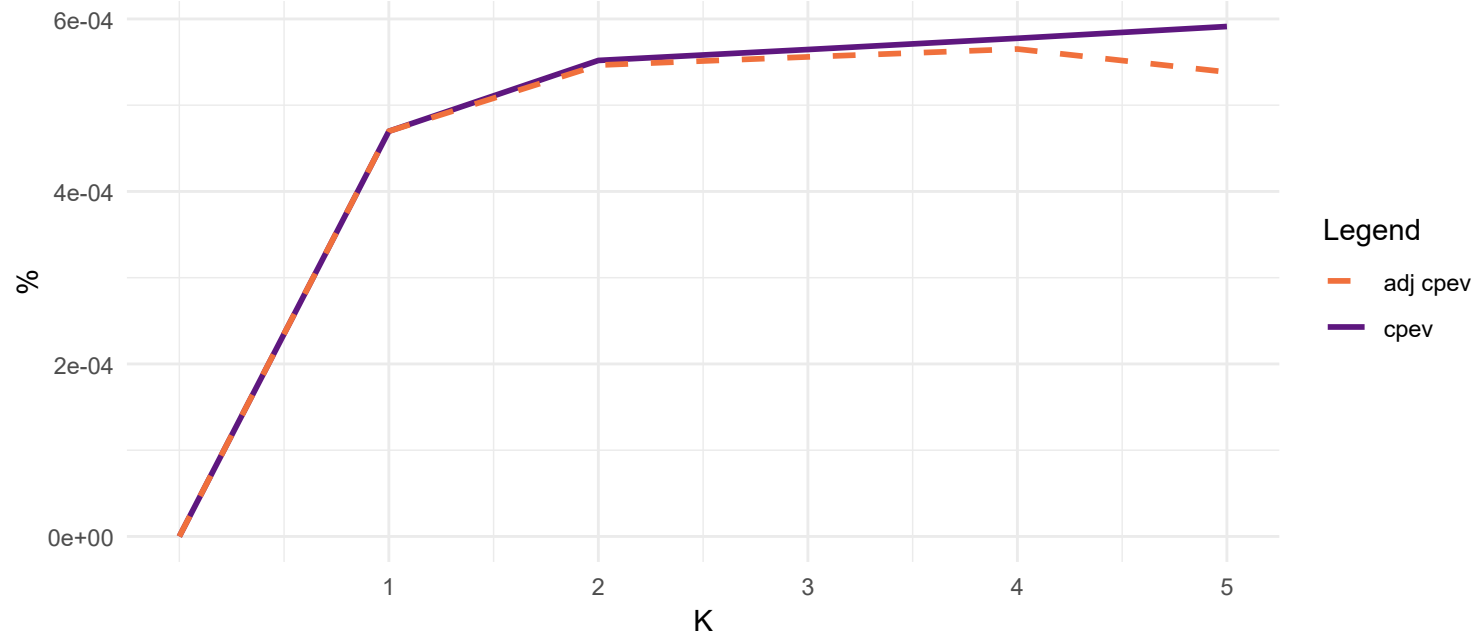

Supplement: Supplementary file 1 — Supporting Information [file BIMJ-67-e70090-s001.zip › tosccammCode_senar/toscca-mm-main/figures/figure_12.pdf]

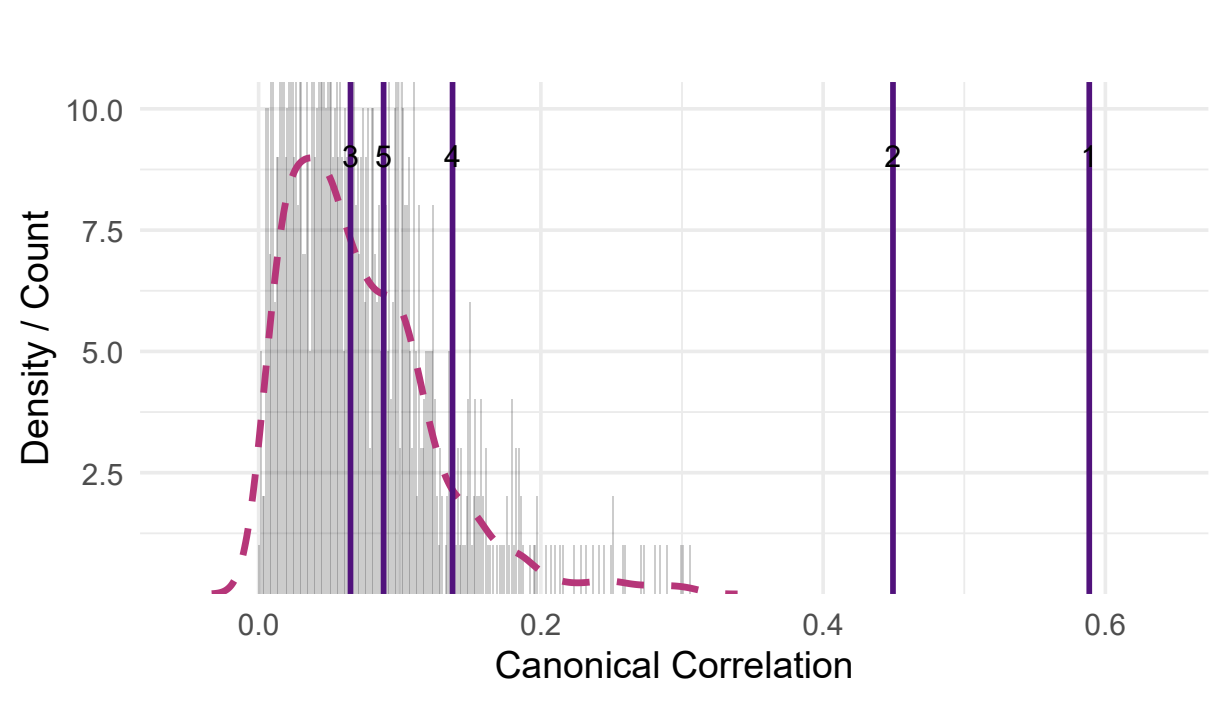

Supplement: Supplementary file 1 — Supporting Information [file BIMJ-67-e70090-s001.zip › tosccammCode_senar/toscca-mm-main/figures/figure_13.pdf]

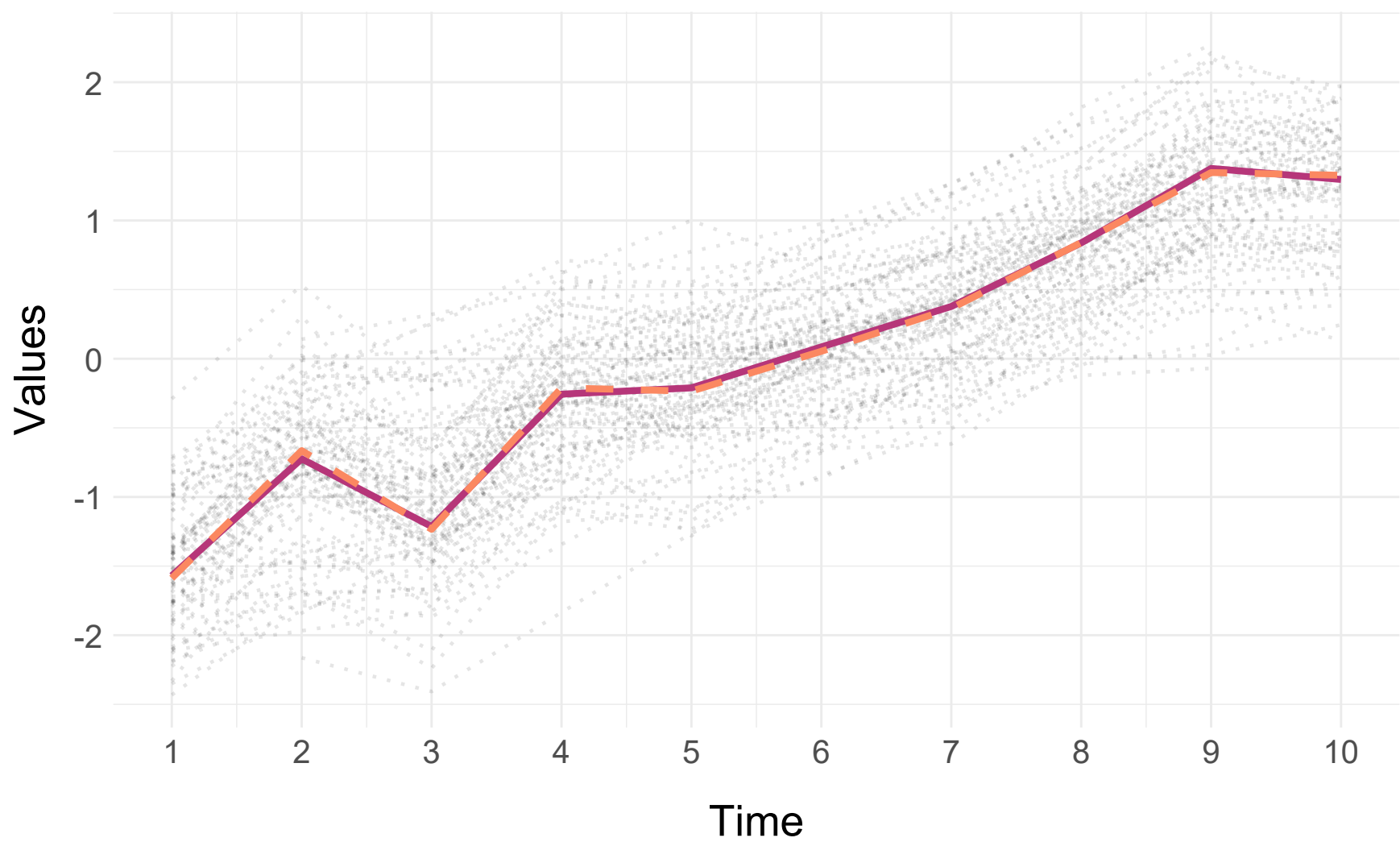

Supplement: Supplementary file 1 — Supporting Information [file BIMJ-67-e70090-s001.zip › tosccammCode_senar/toscca-mm-main/figures/figure_3_a.pdf]

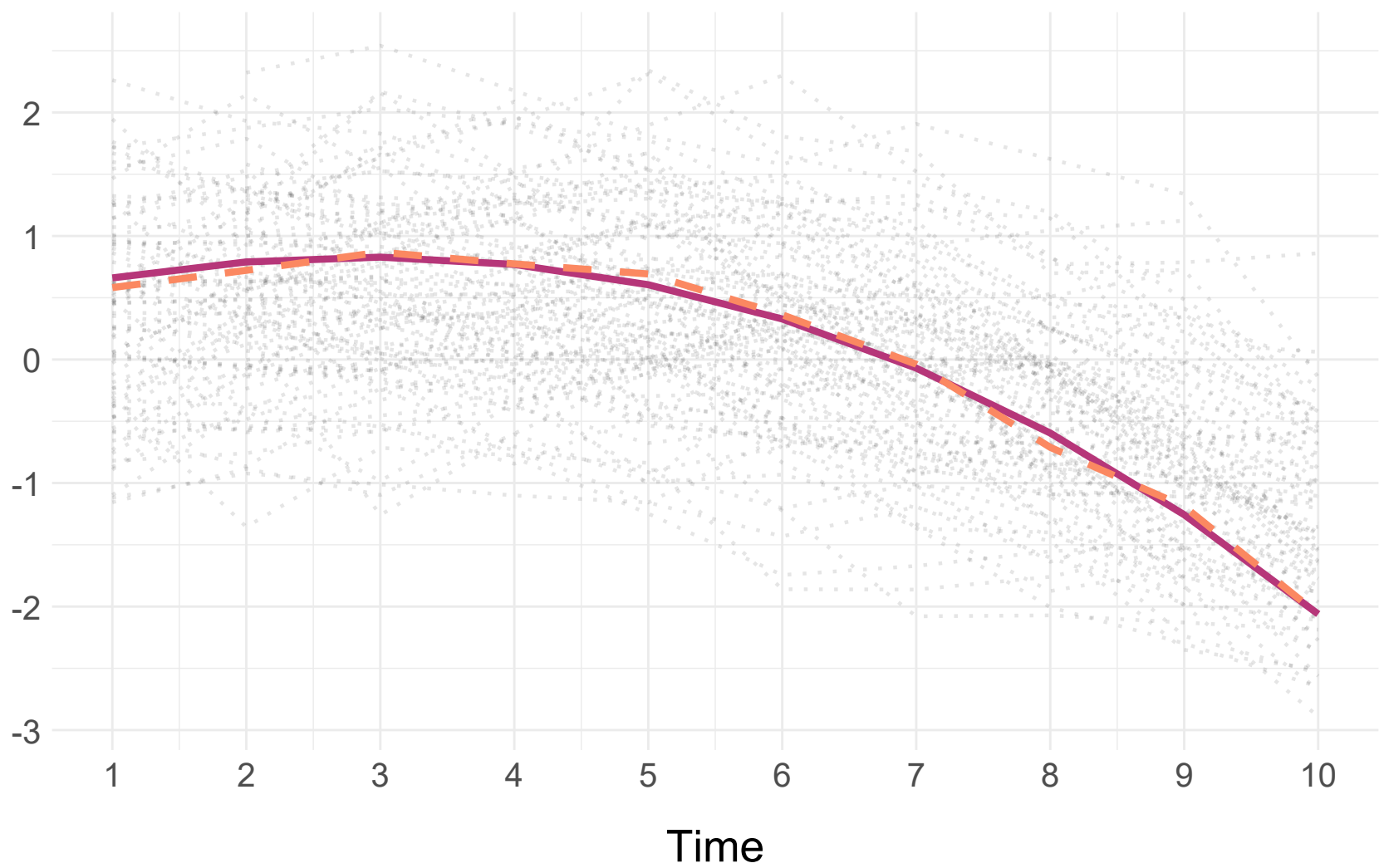

Supplement: Supplementary file 1 — Supporting Information [file BIMJ-67-e70090-s001.zip › tosccammCode_senar/toscca-mm-main/figures/figure_3_b.pdf]

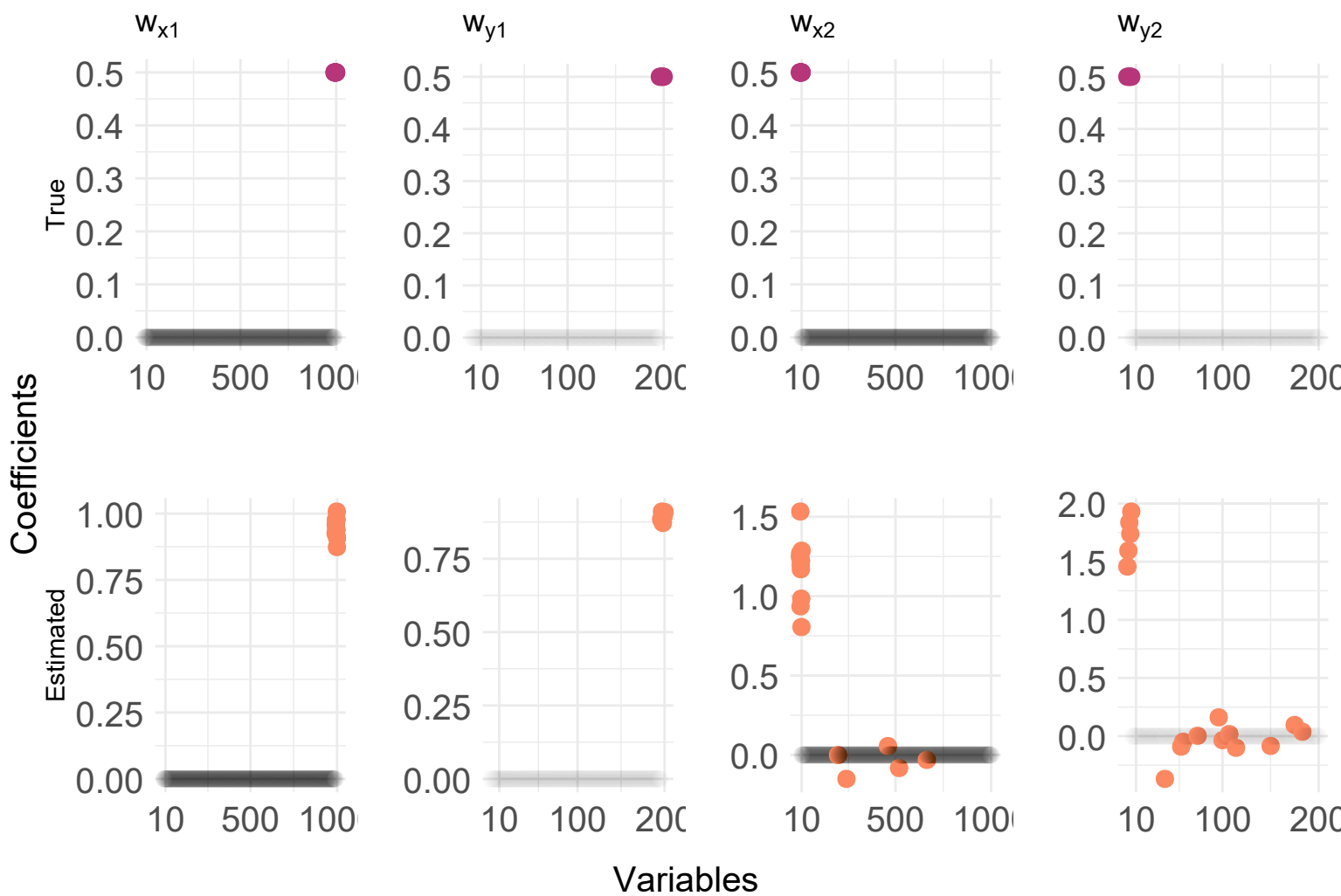

Supplement: Supplementary file 1 — Supporting Information [file BIMJ-67-e70090-s001.zip › tosccammCode_senar/toscca-mm-main/figures/figure_4.pdf]

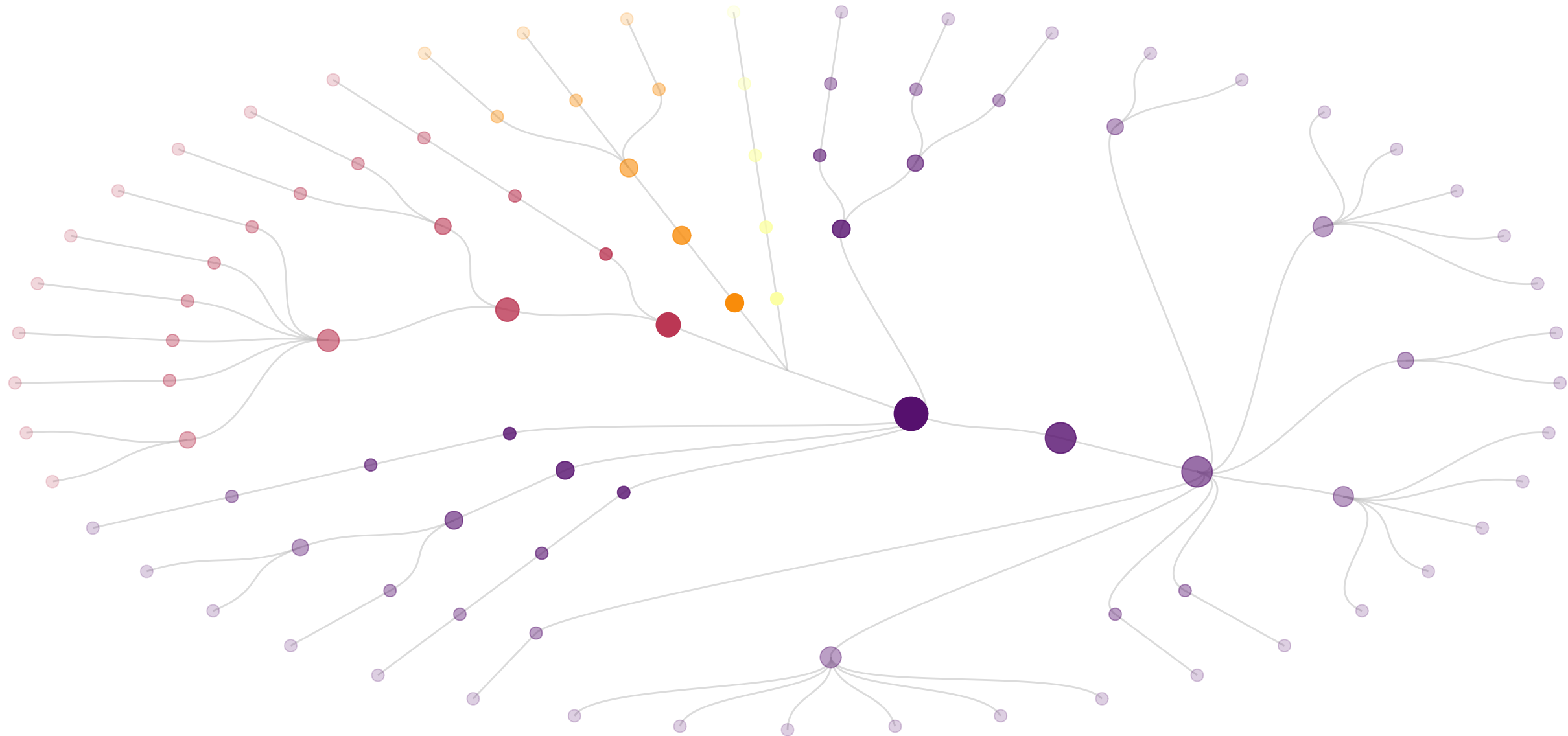

Phylum   Firmicutes   Bacteroidetes   Actinobacteria   Thermodesulfobacteriota

Supplement: Supplementary file 1 — Supporting Information [file BIMJ-67-e70090-s001.zip › tosccammCode_senar/toscca-mm-main/figures/figure_5.pdf]

# cpev toscca

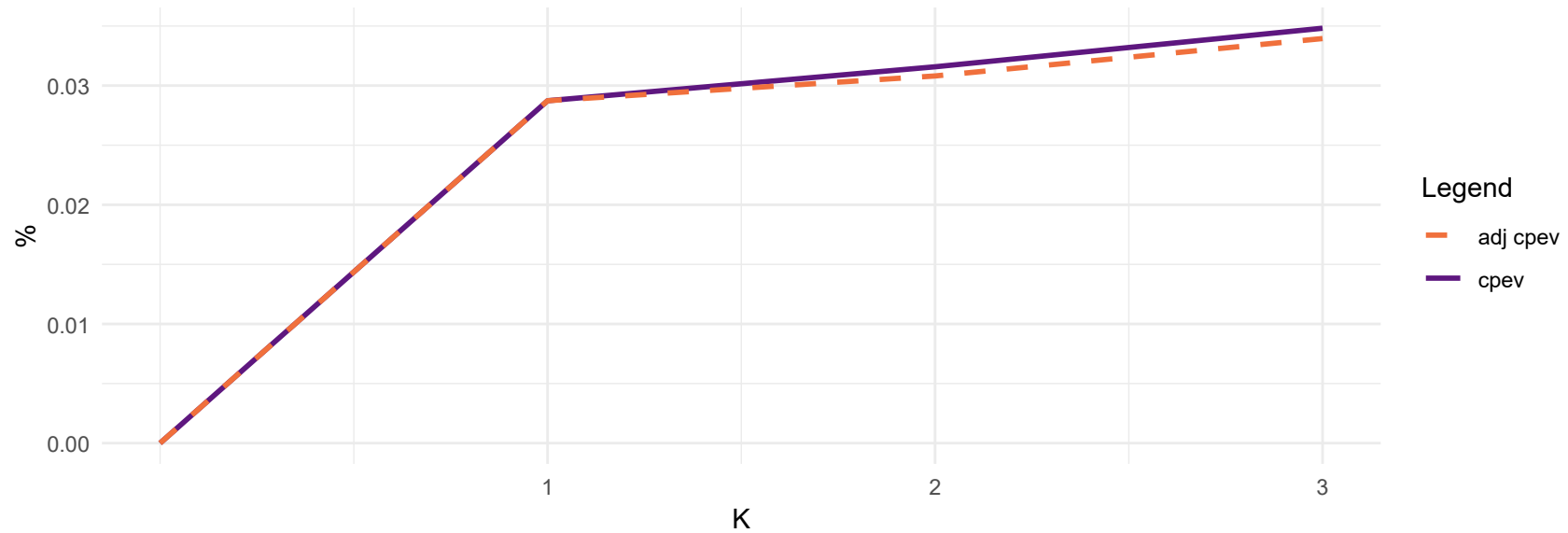

Supplement: Supplementary file 1 — Supporting Information [file BIMJ-67-e70090-s001.zip › tosccammCode_senar/toscca-mm-main/figures/figure_8.pdf]

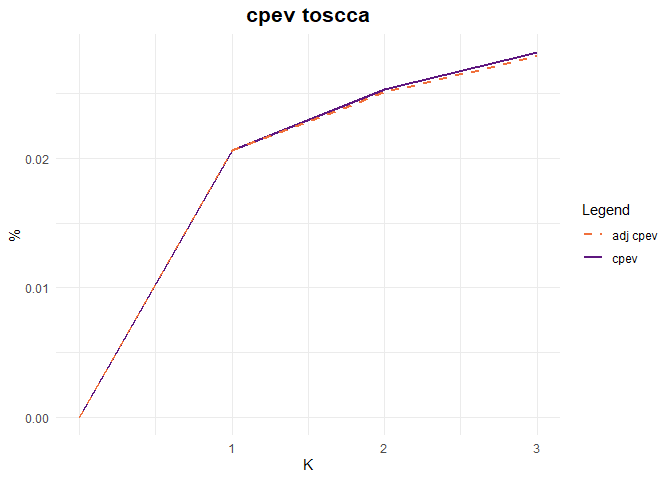

Supplement: Supplementary file 1 — Supporting Information [file BIMJ-67-e70090-s001.zip › tosccammCode_senar/toscca-mm-main/man/figures/README-cpev hmp data-1.png]

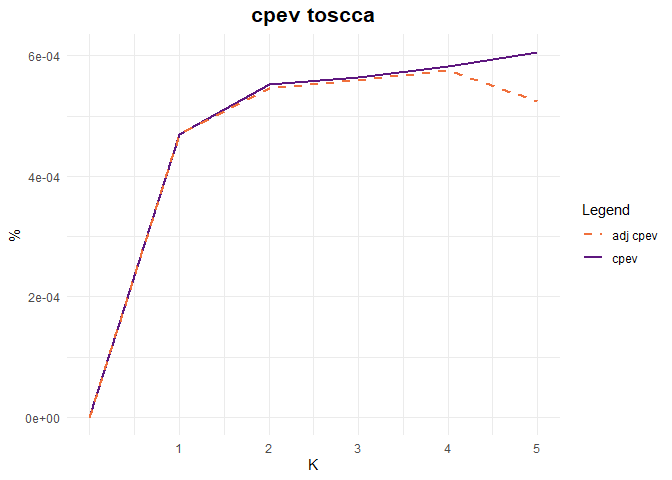

Supplement: Supplementary file 1 — Supporting Information [file BIMJ-67-e70090-s001.zip › tosccammCode_senar/toscca-mm-main/man/figures/README-cpev-1.png]

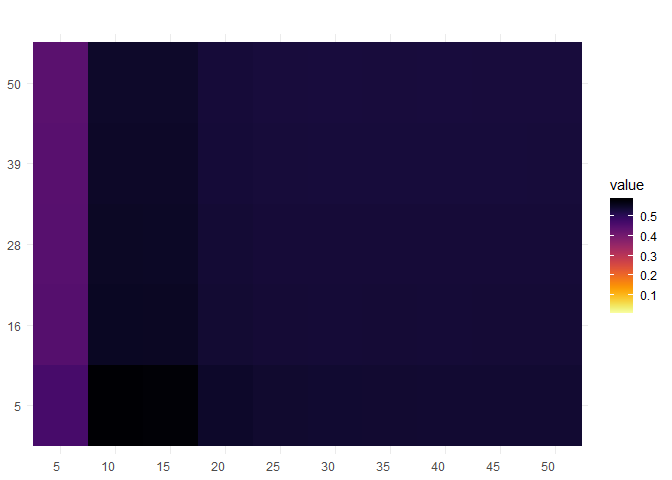

Supplement: Supplementary file 1 — Supporting Information [file BIMJ-67-e70090-s001.zip › tosccammCode_senar/toscca-mm-main/man/figures/README-gridPlots-1.png]

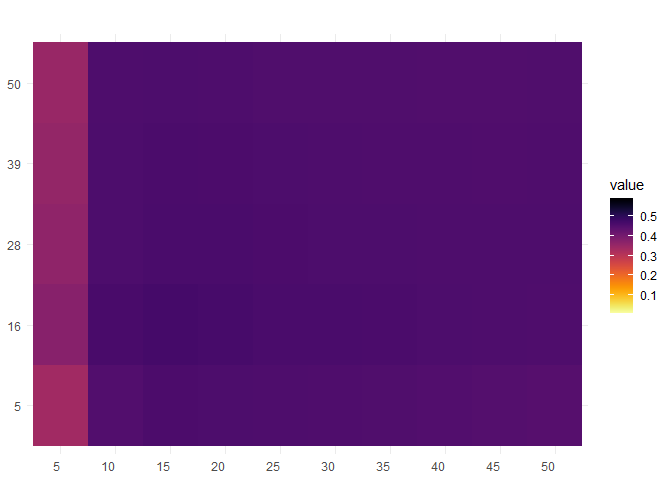

Supplement: Supplementary file 1 — Supporting Information [file BIMJ-67-e70090-s001.zip › tosccammCode_senar/toscca-mm-main/man/figures/README-gridPlots-2.png]

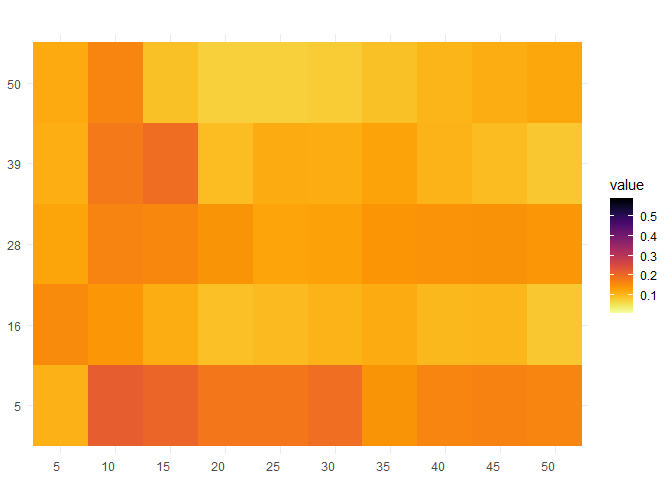

Supplement: Supplementary file 1 — Supporting Information [file BIMJ-67-e70090-s001.zip › tosccammCode_senar/toscca-mm-main/man/figures/README-gridPlots-3.png]

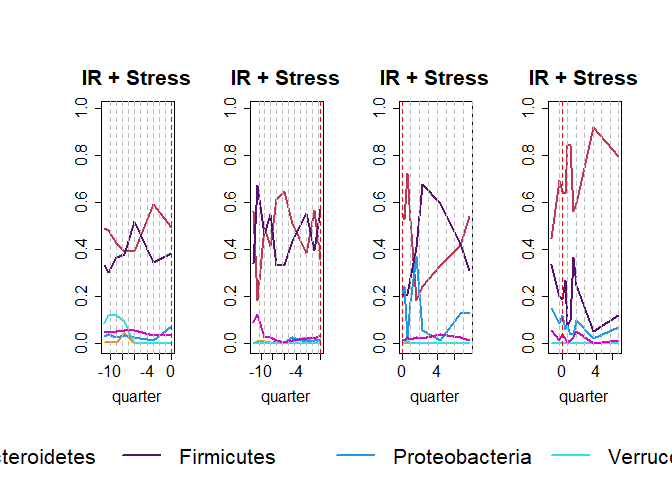

Supplement: Supplementary file 1 — Supporting Information [file BIMJ-67-e70090-s001.zip › tosccammCode_senar/toscca-mm-main/man/figures/README-healthy-1.png]

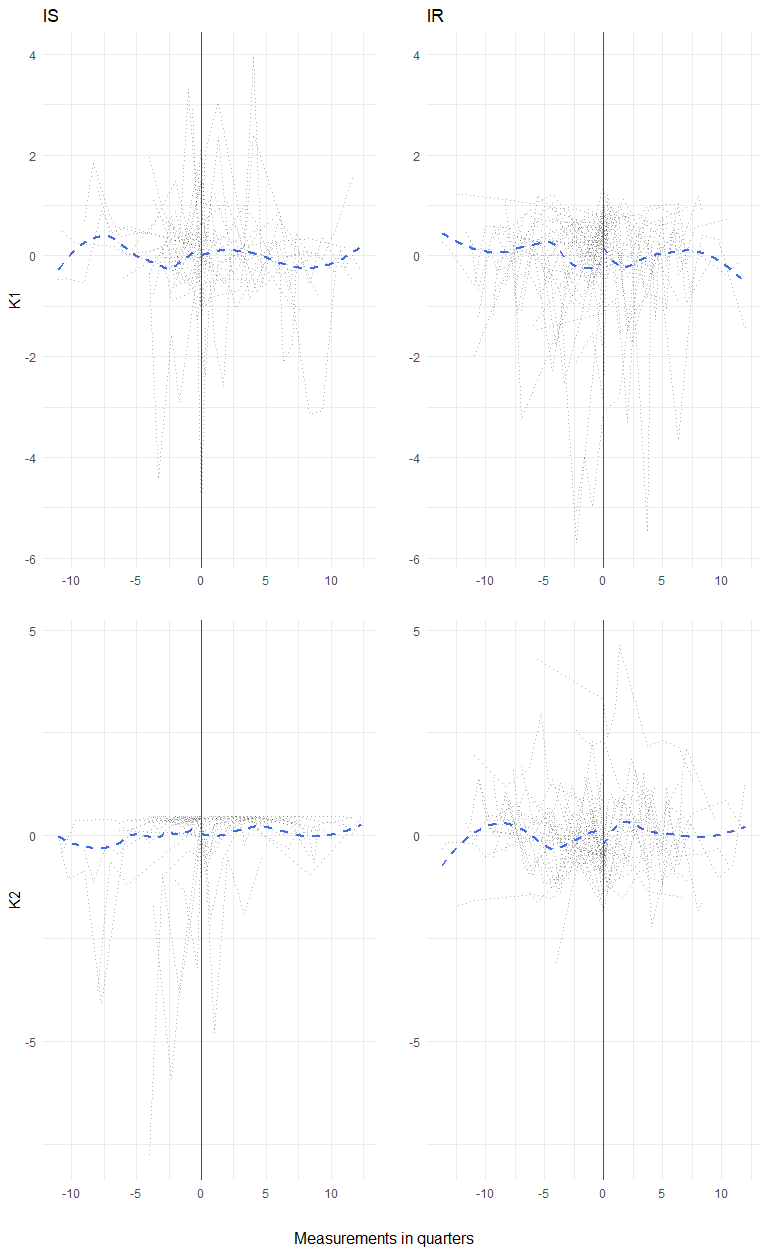

Supplement: Supplementary file 1 — Supporting Information [file BIMJ-67-e70090-s001.zip › tosccammCode_senar/toscca-mm-main/man/figures/README-hmp plots lv class-1.png]

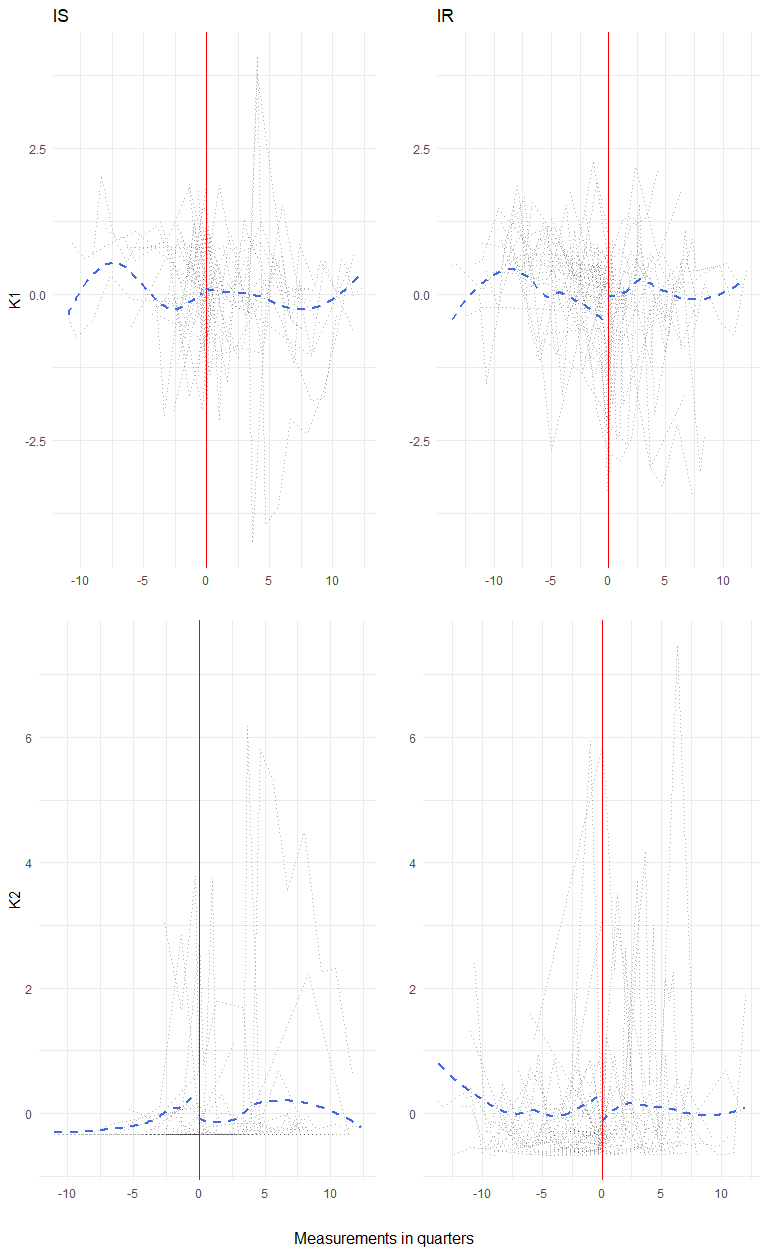

Supplement: Supplementary file 1 — Supporting Information [file BIMJ-67-e70090-s001.zip › tosccammCode_senar/toscca-mm-main/man/figures/README-hmp plots lv fam-1.png]

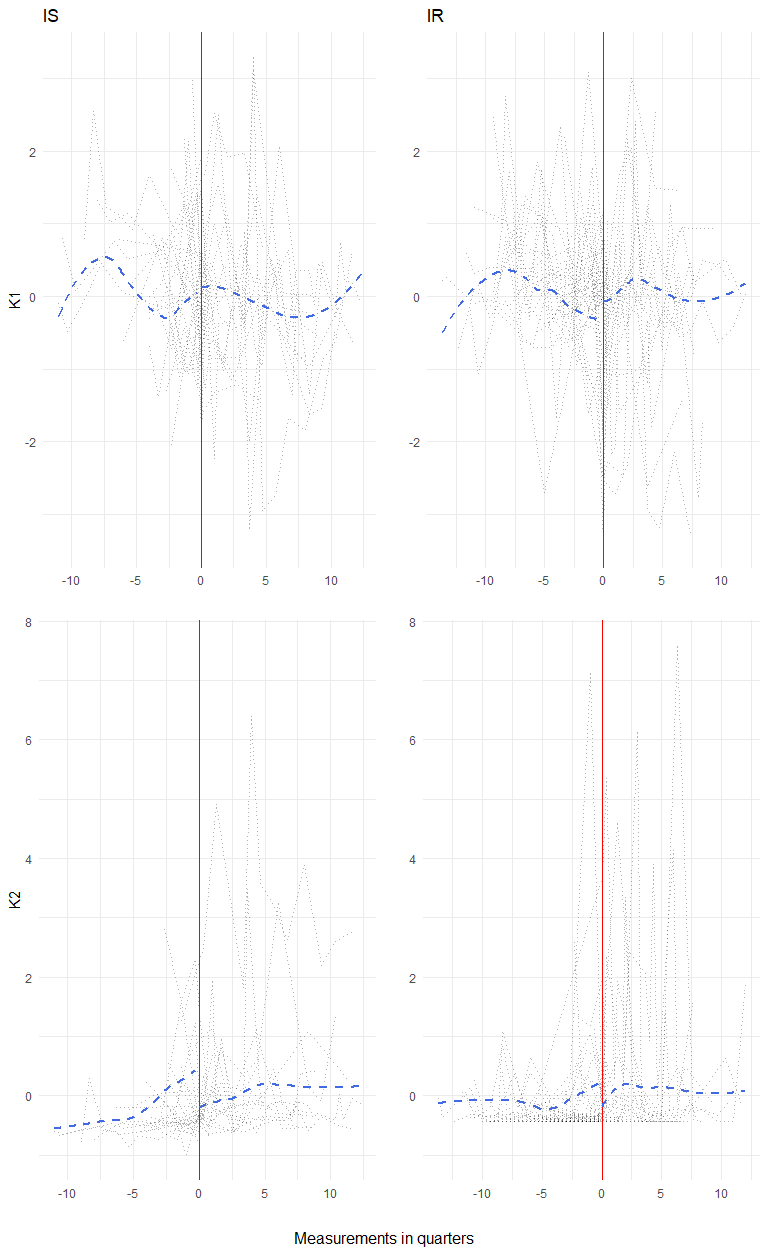

Supplement: Supplementary file 1 — Supporting Information [file BIMJ-67-e70090-s001.zip › tosccammCode_senar/toscca-mm-main/man/figures/README-hmp plots lv genus-1.png]

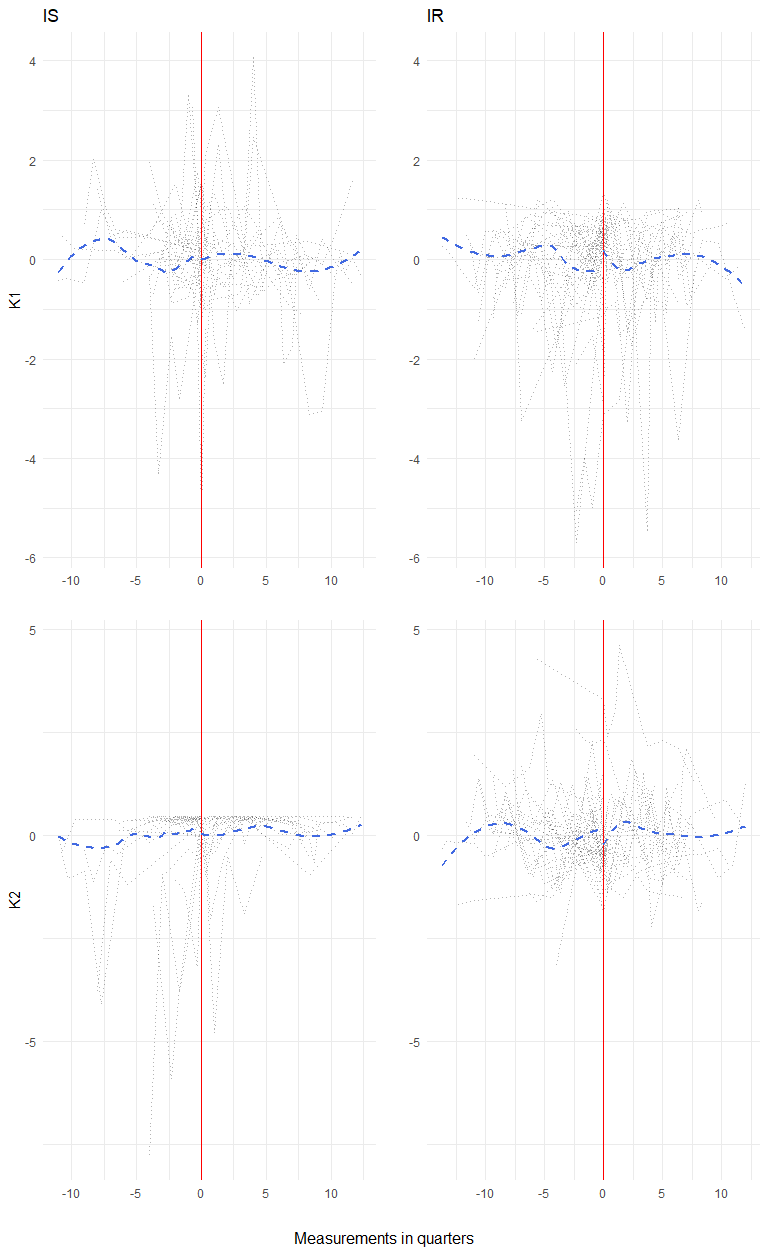

Supplement: Supplementary file 1 — Supporting Information [file BIMJ-67-e70090-s001.zip › tosccammCode_senar/toscca-mm-main/man/figures/README-hmp plots lv order-1.png]

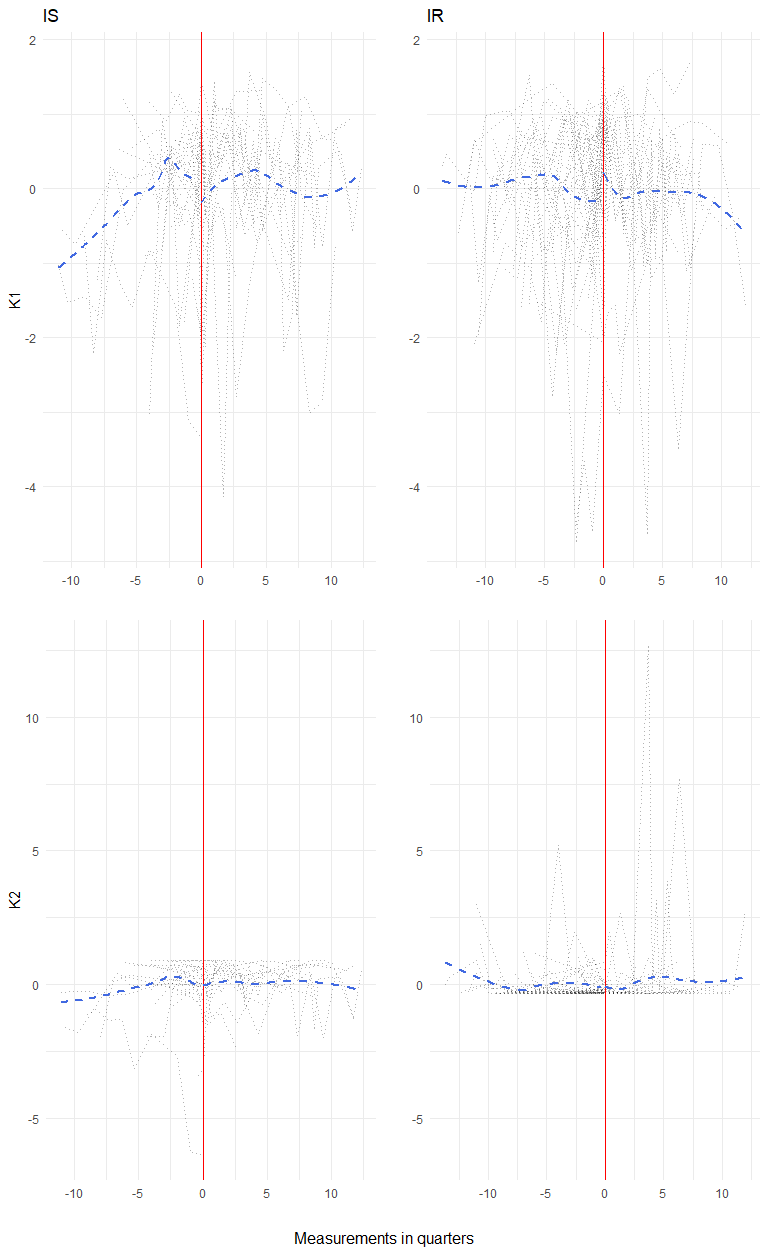

Supplement: Supplementary file 1 — Supporting Information [file BIMJ-67-e70090-s001.zip › tosccammCode_senar/toscca-mm-main/man/figures/README-hmp plots lv phylum-1.png]

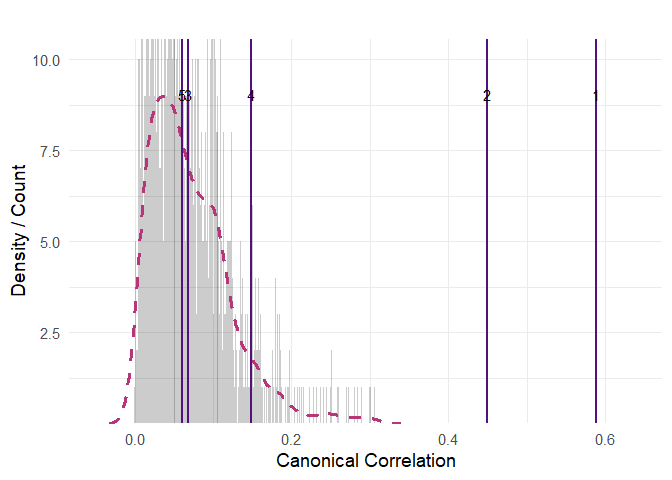

Supplement: Supplementary file 1 — Supporting Information [file BIMJ-67-e70090-s001.zip › tosccammCode_senar/toscca-mm-main/man/figures/README-permutationTesting-1.png]

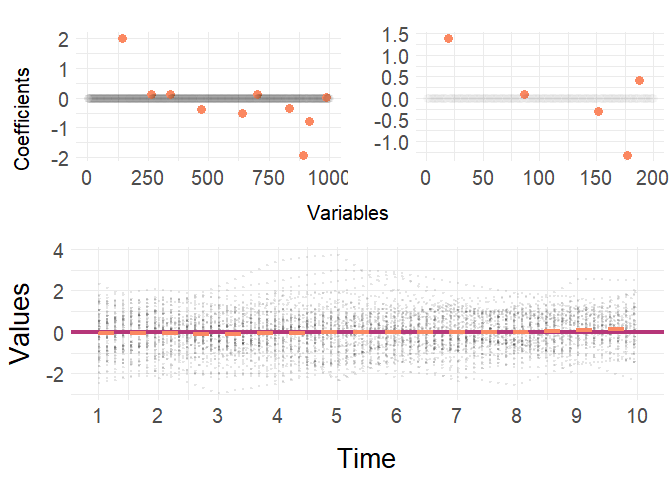

Supplement: Supplementary file 1 — Supporting Information [file BIMJ-67-e70090-s001.zip › tosccammCode_senar/toscca-mm-main/man/figures/README-plotNoise-1.png]

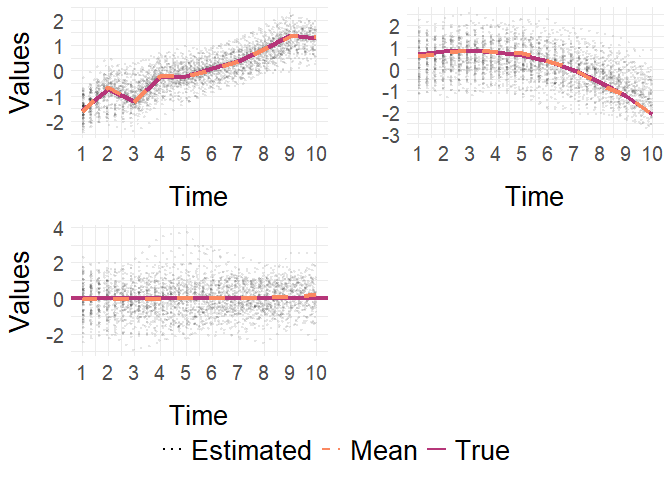

Supplement: Supplementary file 1 — Supporting Information [file BIMJ-67-e70090-s001.zip › tosccammCode_senar/toscca-mm-main/man/figures/README-plots-1.png]

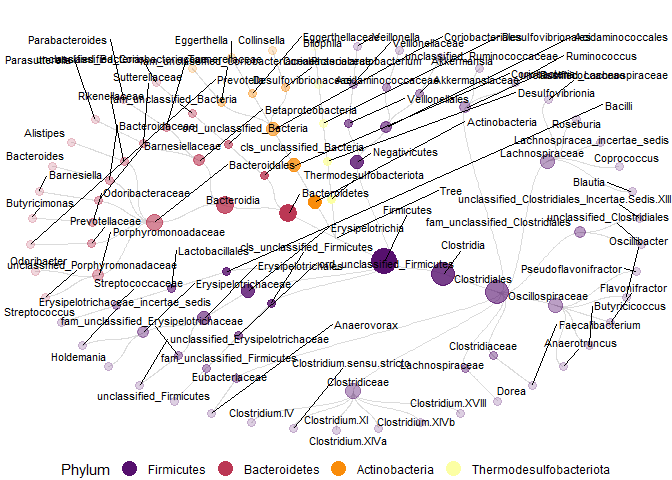

Supplement: Supplementary file 1 — Supporting Information [file BIMJ-67-e70090-s001.zip › tosccammCode_senar/toscca-mm-main/man/figures/README-taxonomic tree-1.png]

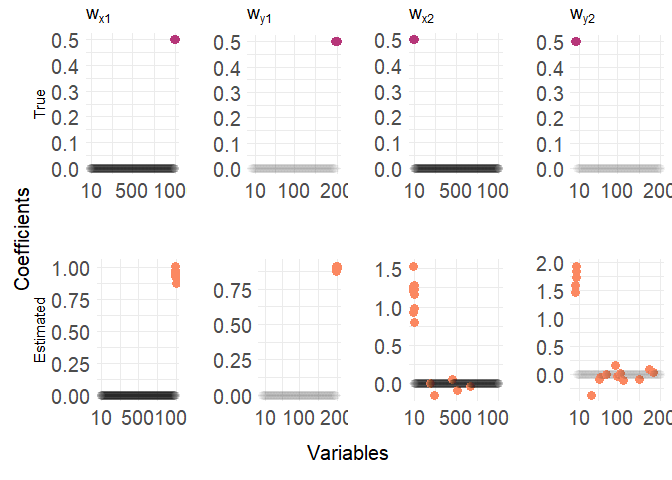

Supplement: Supplementary file 1 — Supporting Information [file BIMJ-67-e70090-s001.zip › tosccammCode_senar/toscca-mm-main/man/figures/README-unnamed-chunk-2-1.png]
